# Novel methods for selecting stock portfolio in conditions of uncertainty and forecasting with RR-DEA, ANFIS, FGP: A case study of Tehran stock exchange

**DOI:** 10.1371/journal.pone.0321370

**Published:** 2025-07-15

**Authors:** Mohammadmahdi Taheri, Amir Azizi, Emran Mohammadi, Abbas Saghaei

**Affiliations:** 1 Department of Industrial Engineering, Science and Research Branch, Islamic Azad University, Tehran, Iran; 2 School of Industrial Engineering, Iran University of Science and Technology, Tehran, Iran; University of Bonab, IRAN, ISLAMIC REPUBLIC OF

## Abstract

Portfolio selection and management are two of the most important decisions in the financial field. The existence of uncontrollable factors affects the decision-making process, which is a problem for investors who are responsible for the final financial decisions on how to allocate their budgets to financial assets in their investment portfolios. To overcome the challenges involved in the selection of a stock portfolio, this article presents a three-stage optimization model. In the first stage, the pharmaceutical industry data collected from the Tehran Stock Exchange (TSE) website is used to apply the robust ratio data envelopment analysis (RR-DEA) in GAMS software with respect to some specific financial indicators to determine efficient stocks in conditions of data uncertainty. These selected stocks are then moved to the second stage, where the ANFIS algorithm is employed in MATLAB to predict the final closing prices and calculate the prediction error (RMSE). In the third stage, the fuzzy goal programming (FGP) method is applied, incorporating the prediction errors from the previous stage. The model is optimized in GAMS software, considering each Index’s objectives in a fuzzy context, with the results presented separately for different objectives. For this problem, in the first stage 27 stocks were selected as samples from the (TSE) website using the proposed methods, and 23 stocks were entered into the price prediction stage. Finally, in the FGP stage, optimization and purchase amount of each share was done. Illustrative results show that the proposed approach is effective for portfolio selection and optimization in the presence of uncertain data.

## 1. Introduction

The portfolio optimization and selection problems are two of the main criteria of studies in investment management. Stock portfolio management emerged from the groundbreaking work of Markowitz [1] through the introduction of the mean–variance method for asset allocation. The classic mean-variance method primarily focuses on two branches, risk and return. Empirical evidence shows that incorporating more than two factors in order to choose the best stock portfolio mitigates reliance on any single measure that might have flaws associated with it [2–7]. Furthermore, contemplating factors beyond return and risk is not an unsound move in a portfolio optimization problem. It may facilitate a reduction in management distraction, as well as resulting in possible improvements to other favorable attributes [8]. Consequently, diversified criteria for stock portfolio optimization and management align with various investment strategies that primarily rely on investor preferences. However, the decision to select a portfolio of stocks and purchase a stock can be more difficult since many attributes must be considered simultaneously. Some of these attributes may include the rate of liquidity, the rate of return, systematic and non-systematic risk, financial ratios, etc. Investors and decision-makers can use the multicriteria decision- making approach to consider more than two criteria in selecting stocks [9–14].

In the context of stock portfolio selection, fundamental analysis is a way of evaluating a company’s investment potential. Fundamental analysis involves a thorough examination of a company’s financial statements until it assesses its investment worthiness, while technical analysis relies on historical trajectories to predict future stock prices [15,16]. Pätäri et al [17]. used the constant returns-to-scale, super efficiency, and cross-efficiency data envelopment analysis methods as a basis of selection criteria for equity portfolios, aiming at integrating the benefits of both value investing and momentum investing. Prior research supports the integration of fundamental analysis and technical analysis, indicating that these two techniques could be complements, rather than substitutes [18–20]. In fact, in the domain of stock portfolio selection, the return and risk attributes of the investment are considered technical criteria. On the other hand, there are various methods to implement fundamental analysis, one of which is Data Envelopment Analysis. Data Envelopment Analysis is a data-enabled performance evaluation technique that evaluates the relative efficiency of decision-making units considering many inputs and outputs and can be leveraged in the process of financial portfolio construction to measure assets’ efficiencies, thereby specifying the best assets for investment [21].

The multi-objective programming technique of Goal programming (GP) is a prominent and powerful MCDM approach that has been applied in various areas of financial decision-making [22]. Multicriteria decision-making approaches have the potential to enable the consideration of various criteria in the stock portfolio selection problem [23]. GP is an instrumental tool for analyzing portfolio selection problems and achieving reasonable solutions concerning the inclusion of the decision-maker’s preferences. In this way, they are able to benefit from the capabilities of this mathematical framework, including sensitivity analysis, to effectively achieve their desired stock portfolios. Stock portfolio optimization problems concentrate only on return, and risk optimization can be described as a GP model comprising two principal ideals.

Another point that should be considered in the proposed approach for portfolio construction is the uncertain nature of parameters. Uncertainty of financial markets is one of their most essential characteristics [24]. Investment decisions should be made by incorporating adequate hedging against uncertainty; otherwise, the resultant portfolios would not be practical and reliable for real-world applications.

In the portfolio selection literature, robust optimization (RO) and stochastic programming (SP) are two factors of the widely-adopted methods utilized to cope with the uncertainty. Both RO and SP seek to address the same question of building an uncertainty–immunized solution to a portfolio selection problem. When the uncertain data are stochastic, the quality of SP-based decisions is associated with the type of underlying probability distribution [25,26]. Considering the aforementioned drawbacks associated with RO, SP, on the other hand, utilizes uncertainty sets instead of probability distributions to account for uncertain data and does not cause intractability [27]. Ahmed et al. [28] examined risk management (RM) practices. For this purpose, Insurance personnel on risk understanding and risk management (URRM), risk identification (RI), risk assessment and analysis (RAA), risk monitoring (RMON), and risk management practices (RMP) were examined. The results of their work showed that (RM) Insurers are stronger, but there are many differences at the hierarchical level, the type of insurer, and the level of the country. Also, their findings showed that to achieve sustainable competitive advantage, insurers should minimize these differences. Akram et al. [29] investigated the relationship between green growth and traditional economic growth. In their study, they investigated the impact of financial fragility and the influence of information and communication technology on renewable energy consumption and green growth for the top five polluting economies during the period from 1996 to 2020. Their findings showed policy implications for the green economy. Khelfaoui et al. [30] investigated the impact of education on health shocks, whether these health shocks are subjective or objective. In order to estimate the causal relationship between education and health, Chinese health and nutrition surveys have been investigated through linear regression and multi-logic models, respectively. The results show that higher education has a positive and significant effect on health shocks, regardless of subjective or objective shock. Ahmed et al. [31] examine the impact of investor sentiment on non-life insurance demand during economic downturns. They used the original dataset of 33 countries’ OECD from 2007 to 2016 and used the bias-corrected bootstrapping technique to create a large dataset with 10,220 observations. They argued that large datasets from the bias-corrected bootstrapping technique produce unbiased and efficient regression estimates. The results show that during periods of economic decline, risk-averse people buy insurance policies to preserve their wealth, and as a result, the demand for non-life insurance increases.

## 2. Literature review

This article is connected to three areas of literature. First, it explores the application of data envelopment analysis (DEA) in financial decision-making and portfolio selection. Next, it introduces the use of the ANFIS algorithm for financial forecasting. Finally, the article delves into the literature surrounding fuzzy goal Programming within the context of finance.

### 2.1. Contextual background

#### 2.1.1. Applications of DEA in stock portfolio optimization.

DEA methods have been used for efficiency evaluation in many fields like education, athletics, finance, economics, and so on [32]. DEA methods have been used as a means for efficient estimation of post-creation stock portfolios. As such, different risk measures, like the variance of the stock portfolio, have been used as inputs, and the expected return of the stock portfolio has been used as the output of these approaches [33–35]. Peykani et al. [36,37] proposed a two-phase method for the stock portfolio optimization and selection problems in which robust DEA methods were used in the first stage to measure stock efficiency and select candidate assets for the stock portfolio selection stage in the presence of uncertain data. They then applied single-goal robust mean absolute deviation-liquidity and robust mean-semi variance-liquidity methods to determine candidate investment weights. They applied their models to the Tehran Stock Exchange (TSE). Amin and Hajjami [38] developed a cross-efficiency DEA method for the stock portfolio optimization problem. They found that stock portfolio construction with higher returns and lower risk is possible when alternative optimal solutions are included in the method. In an intention to incorporate data mining methodology into the stock portfolio optimization and selection plan, Zhou et al. [39] proposed a stock optimization plan in multiple data sources with integrated DEA, and then used a support vector machine to predict the stock price movements and combined it with the stock optimization scheme to construct the stock portfolio selection method. Henriques et al. [40] proposed a two-stage approach for stock portfolio optimization, utilizing DEA to identify efficient assets based on financial criteria and interval multi-objective stock portfolio methods to determine optimal portfolio compositions according to investor preferences. More recent applications of DEA in portfolio selection can be found in the work of Hosseinzadeh et al. [41], where the authors employed an SBM DEA model to preselect efficient assets in large-scale stock portfolio problems. They introduced different reward/risk criteria and DEA input/output sets based on these criteria for asset preselection, subsequently evaluating the impact of preselected assets on stock portfolio selection problems. Li et al. [42] present a two-objective portfolio model based on uncertainty and a BCC-DEA model for evaluating high-risk portfolios. To evaluate the presented models, some numerical simulations were presented to show the effectiveness and practicality of the models based on the double-objective genetic algorithm.

#### 2.1.2. ANFIS.

The ANFIS method was introduced by Jang [43] as a novel artificial network (ANN). The ANFIS method’s structure is considered the incorporation of Fuzzy Inference Systems (FIS) and ANN. In addition, “IF-THEN rules” are applied to generate a mapping for the inputs and outputs, identified as the “Takagi–Sugeno inference method.” This substantiates that the ANFIS method is reliable and more convenient for dealing with real global datasets as it has a robust learning capability. As stated by these characteristics, the ANFIS method has been implemented in many applications.

**Fig 1:**
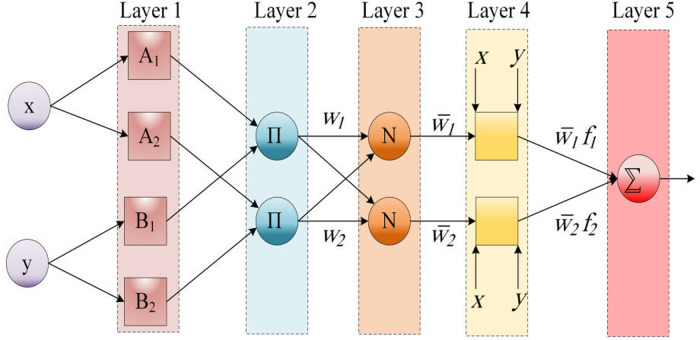
The basic ANFIS structure.


In the common ANFIS workflow, as drawn in Fig 1, x and y show the inputs of layer 1, where O_1i_ indicates the outputs of the i node. The ANFIS mathematical method is expressed as follows:

$$O_{1i}\;=\;\mu_{Ai}\;(x),\;i\;=\;1,\;2,\;O1i\;=\;\mu_{Bi-2},\;i\;=\;3,\;4$$ (1)

$$\mu (x)\;=\;e^{{-(\frac{x-\rho_{i}}{\alpha_{i}})}^{2}}\;;$$ (2)

where µ shows the generalized Gaussian membership function. The membership values of µ are represented by A_i_ and B_i_, and α_i_ and ρ_i_ show the premise parameter set. In addition, Equation (3) can be utilized for the second layer:


$$O_{2i}\;=\;\mu_{Ai}\;(x)\;\times \;\mu_{Bi}-2\;(y)$$ (3)

The output of the third layer is calculated as:


$$O_{3i}\;=\;{\overset{―}{w}}_{i}\;=\frac{w_{i}}{\sum_{i=1}^{2}{\overset{´}{w}}_{i}}$$ (4)

In which w_i_ represents the ith output from layer 2. In addition, the output of layer 4 is generated by Equation (5).


$${O_{4,i}\;=\;{\overset{―}{w}}_{i}f_{i}=\overset{―}{w}}_{i}(p_{i}x\;+\;q_{i}y\;+\;r_{i})$$ (5)

In this case, f shows a function that uses the input and parameters of the network as inputs. r_i_, q_i_ and p_i_ show consequent parameters of node i.

Finally, layer 5 generates the output that is computed as in Equation (6).


$$O_{5}=\sum_{i}{\overset{―}{w}}_{i}f_{i}$$ (6)

Esfahanipour and Aghamiri [44] proposed a Neuro-Fuzzy Inference System adopted on a Takagi-Sugeno-Kang (TSK) type Fuzzy Rule-Based System developed for stock price prediction. The proposed method is tested on the Tehran Stock Exchange Indexes (TEPIX). This Index, with high accuracy, has been successfully forecasted using several experimental tests from different indicators. Bagheri et al. [45] present a novel hybrid intelligent model to forecast financial time series for the Foreign Exchange Market (FX). The second part of this paper proposes a new hybrid Dynamic Time Warping (DTW)-Wavelet Transform (WT) model for automatic pattern extraction. The results show that the presented hybrid model is beneficial and effective for financial pattern extraction and financial price forecasting. Gunasekaran et al. [46] address a method that suggests stock portfolio optimization using the Capital Asset Pricing Model (CAPM) and the combination of the Adaptive Neuro-Fuzzy Inference System (ANFIS). Experimental results show that the proposed hybrid intelligent system ANFIS-CAPM yields better performance than existing portfolio models. Chanchal Kumar et al. [47] propose a new framework for solving the portfolio selection problem. This framework is excogitated using two new parameters obtained from an existing basic mean- variance model. Finally, The numerical results acquired from the framework and the new structure are presented, and these results are assimilated and compared with the results of the existing ANFIS structure. Yuxuan Huang et al. [48] present a comparative study that investigates and compares feed-forward neural networks (ANN) on stock prediction using fundamental financial ratios. The study is designed to evaluate the performance of each architecture based on the relative return of the selected portfolios with respect to the benchmark stock index. The results indicate that both architectures possess the ability to separate winners and losers from a sample universe of stocks, and the selected portfolios outperform the benchmark. Their study argues that FNN indicates superior performance over ANFIS. Ghahtarani et al. [49] propose a portfolio selection method developed using a new risk measure. The proposed risk measure is based on the fundamental value of stocks. For this goal, a mathematical method is developed and transformed into an integer linear programming. Data mining models which are used in this article include ANFIS, decision tree, random forest, ADF, and GEP. The best method for scenario evaluation is GEP based on numerical results. Wuzhida Bao et al [50] present comprehensively review the literature on data-driven neural networks in the field of stock forecasting from 2015 to 2023, discussing innovative neural network structures and various classics, including Recurrent Neural Networks (RNN_S_), Convolutional Neural Networks (CNN_S_), Transformers, Graph Neural Networks (GNN_S_), Generative Adversarial Networks (GAN_S_), and Large Language Models (LLM_S_). Cao et al. [51] present a simplified model for portfolio management optimization in quadratic planning mode and developed the Markowitz model. For this purpose, this model decomposes the problem into solvable and unsolvable analytical components. On the other hand, a dynamic neural network is also designed to quickly solve unsolvable components. Finally, in an experiment using stock data (DJIA), the presented model reduced the total costs by 5.54% compared to other solvers.

#### 2.1.3. Goal programming in stock portfolio optimization.

Goal programming is a practical model for analyzing stock portfolio optimization problems with several conflicting and incommensurable criteria, allowing for the generation of plausible solutions that align with the FDM’s preferences. Depending on the nature of the available information, the FDM is required to select the suitable GP variant to derive a solution that best aligns with their stock portfolio optimization preferences.

Tamiz et al [52,53] applied three goal programming variants- Lexicographic, Weighted, and MinMax GP to develop stock portfolio optimization methods for international mutual funds, which enable decision-makers to incorporate their preferred criteria and goal aspiration levels into the GP method for acquiring their intended stock portfolio. The authors selected seven criteria from 3 classifications: mutual funds specific criteria, factors for regional preferences, and macroeconomics criteria. Each factor was treated as a goal in their GP methods. Using data related to 20 mutual funds of equities from 10 various countries, they compared the resulting portfolios with various combinations of priority levels, target values, and weights in the GP methods against each other in terms of risk, return, and the number of mutual funds selected, and demonstrated and discussed the applicability of their approach. Messaoudi et al. [54] introduced a fuzzy chance-constrained GP method to solve a stock portfolio optimization problem with three factors. In their model, stochastic uncertainty pertains to the independent chance-constrained goals, and the financial decision maker’s preferences were considered as uncertain values. De et al. [55] applied a fuzzy GP method using Werner’s ‘fuzzy and’ hybrid operator for a stock portfolio optimization problem with three factors: return, risk, and liquidity. They assumed liquidity and return as fuzzy values and described them with trapezoidal and triangular membership functions. Tamiz and Azmi [56] utilized 5 FA-based criteria, in addition, to return and risk, for stock portfolio optimization, with each factor represented as an ideal in a weighted goal programming (WGP) method. Different WGP methods with various combinations of weights and target values were developed. They applied their methods to thirty stocks from the Dow Jones Industrial Average index, and the resulting portfolios were compared against each other, as well as against well-known benchmark methods for stock portfolio optimization from the literature. The results obtained supported the use of criteria utilized besides return and risk, referred to as extended criteria,’ for addressing stock portfolio optimization problems. Mansour et al. [57] formulated a GP method for the stock portfolio optimization problem with three factors: return, risk, and liquidity. In this method, uncertainty returns were assumed, and investor preferences were incorporated using the concept of satisfaction functions. Deng and Yuan [58] proposed a GP method based on fuzzy dominance for a stock portfolio optimization problem involving fuzzy returns while simultaneously considering non-systematic and systematic risks. More recently, Bravo et al. [59,60] applied a GP method that differs from the state-of-the-art uncertain GP methods. In this way, the variability of parameters resulting from randomness is addressed by replacing the traditional WGP achievement function with a new function that takes into account the decision-makers perception of randomness through the use of a penalty term. They applied their approach to a mean absolute deviation stock portfolio optimization problem and claimed that their model could effectively address the challenges arising from the lack of statistical information about random events. Stoyan and Kwon [61] proposed a mixed integer stochastic GP method for bond portfolio problems and an integrated stock, taking into account uncertainty in asset prices and several major trading constraints. Bilbao-Terol et al. [62] proposed MinMax GP and Weighted methods for selecting portfolios with socially responsible investment (SRI)-funds, where they measured the socially responsible performance of financial products using an index built through the application of fuzzy set theory techniques. Mohseny et al. [63] proposed robust extended goal programming with uncertainty sets. In this method, efficient stocks are first selected using the proposed DEA method, and then, considering the uncertainty of the data, the optimization of the stock portfolio is done with the robust goal programming method.

### 2.2. Research gaps

- Uncertainty in financial invoice data for each share
- Forecasting the price of each share and reducing the forecasting error
- Stock portfolio optimization with non-deterministic goals

## 3. Methodology

### 3.1. Introduction

After presenting the general outline and literature review, this research will be conducted in three main stages based on the proposed methodology. The purpose of studying stock portfolio optimization in conditions of uncertainty in the data related to each financial factor of each share and predicting the stock price as accurately as possible. The innovation of this study is the use of the proposed methods RR-DEA, the proposed ANFIS algorithm, and the use of fuzzy goal programming.

If the input and output data for Decision-Making Units are accessible, DEA models introduce the efficiency of the units along with the efficiency patterns on the efficiency center. However, suppose the data are solely ratios of input to output data or vice versa, such as total profits in a year to the number of common stocks or the market value of common stocks to the profits per share in the last year of the company. In that case, DEA models cannot determine the efficiency and patterns of the units. Therefore, this research will employ Ratio-based Data Envelopment Analysis (DEA-R) under conditions of data uncertainty in the first stage of modeling to address the problem.

In the second stage, the Enhanced Adaptive Neuro-Fuzzy Inference System (ANFIS) is utilized to predict stock prices with high precision so that it can cover the inaccuracies from the range of different factors impacting stock prices. The modeling process will attempt to enhance the used algorithms, select the most optimal clustering, improve the predictive powers of neural networks, and mitigate Root Mean Square Error (RMSE).

**Fig 2:**
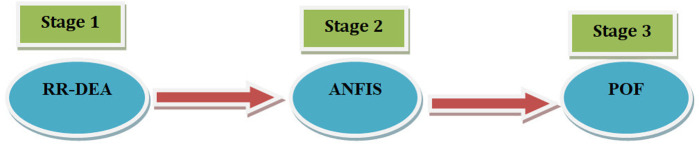
Stages of conducting the research.

The third stage, Portfolio Optimization With Forecasting (POF), involves multi-objective optimization, which utilizes goal programming for portfolio optimization. Here, the forecast errors from ANFIS in stage 2 are incorporated into the third stage so that the stock prices are forecasted based on what has occurred over time *T*. The integration and employment of the stages above have not been done in past research and will be an innovation in this study. In the RR-DEA stage, since the modeling is ratio, there should not be zero data in the data. In the prediction stage with the proposed ANFIS algorithm, if the amount of data is too close to zero, the prediction results may not be favorable. Stages of conducting the research are presented in Fig 2.

### 3.2. Explanation and modeling using the R-DEA method

The most significant Data Envelopment Analysis (DEA) models include the Charnes, Cooper, and Rhodes (CCR) model, the Banker, Charnes, and Cooper (BCC) model, and the Anderson-Peterson model. These models can be employed in various efficiency evaluation problems with the Data Envelopment Analysis (DEA) method. The model of choice dramatically affects the quality of the results; therefore, the choice must be made cleverly. The main approaches are as follows:

Indices:

j set of stocks j=1,…,n

i set of inputs i=1,…,m

r set of outputs r=1,…,s

Parameters:

$x_{ip}$ i^th^ input of stock_p_ (the stock under investigation)

$y_{rp}$r^th^ output of stock_p_ (the stock under investigation)

$x_{ij}$ i^th^ input of j^th^ stock

$y_{rj}$ r^th^ output of j^th^ stock

Decision Variable:

$\backslash {(}_{j}$ weight for the j^th^ stock

**Input-Oriented**–**Envelopment** (CCR-IO)


$$\begin{array}{c} Min\;\emptyset \end{array}$$

$$\begin{array}{c} s.t\left\{ \begin{array}{c} \sum_{j=1}^{n}\lambda jy_{rj}\;{\geq y}_{rp}\;\;\;,\;\;\;\;\;\;r=1,\;\ldots ,s\\ \overset{n}{\underset{j=1}{\sum \lambda }}jx_{ij}\leq \emptyset \;x_{ip}\;\;\;\;\;\;\;,\;\;\;\;\;\;i=1,\;\ldots ,m\; \end{array}\right. \end{array}$$ (7)

$$\begin{array}{c} j\geq 0\;,\;\emptyset \;free\;,\;j=1,\;...,n\; \end{array}$$

**Output-Oriented**–**Envelopment** (CCR – 00)


Max ϕ

$$\begin{array}{c} s_{.}t\;\left\{ \begin{array}{c} \sum_{j=1}^{n}\lambda jy_{rj}\geq \;{\phi y}_{rp}\;\;\;\;\;\;,\;\;\;\;\;\;r=1,\;\ldots .,s\\ \overset{n}{\underset{j=1}{\sum \lambda }}jx_{ij}\leq \;x_{ip}\;\;\;\;\;\;,\;\;\;\;\;\;i=1,\;\ldots .,m\; \end{array}\right. \end{array}$$ (8)

$$\begin{array}{c} \lambda_{j}\geq 0,\;\phi \;free\;,\;j=1,\ldots ,n \end{array}$$

A Decision-Making Unit (DMU) is considered efficient when the objective functions ∅ and  ϕ are equal to one. Otherwise, the DMU is inefficient.

**Additive Model**


$$Max\;\sum_{i=1}^{m}s_{i}^{-}+\;\sum_{r=1}^{s}s_{r}^{+}\;$$

$$s_{.}t\;\left\{ \begin{array}{c} \sum_{j=1}^{n}jx_{ij}\;+s_{i}^{-}=x_{ip}\;,\;i=1,\;\ldots .,m\\ \sum_{j=1}^{n}jy_{rj}-s_{r}^{+}\;=y_{rp}\;,\;r=1,\;\ldots .,s\;\\ \; \end{array}\right.\;$$ (9)

$${\mathrm{\backslash [}}_{j}\;,\;s_{i}^{-}\;,\;s_{r}^{+}\geq 0,\;j=1,\;\ldots .,n\;,\;i=1\;,\;\ldots ,\;m\;,\;r=1,\;\ldots ,\;s$$

A Decision-Making Unit (DMU) is considered efficient when the objective function is equal to zero. Otherwise, the DMU is inefficient.

**Russell Model**


$$Min\;\frac{\frac{1}{m}\;\sum_{i=1}^{m}\emptyset_{i}}{\frac{1}{s}\;\sum_{r=1}^{s}\phi_{r}}$$

$$s_{.}t\;\left\{ \begin{array}{c} \sum_{j=1}^{n}jx_{ij}\;\leq \emptyset_{i}x_{ip}\;,\;i=1,\;\ldots .,m\\ \sum_{j=1}^{n}jy_{rj}\geq \phi_{r}y_{rp}\;,\;r=1,\;\ldots .,s\;\\ \; \end{array}\right.\;$$ (10)

$${\mathrm{\backslash [}}_{j}\geq 0\;,\;j=1,\;\ldots .,n\;$$

$$\;\phi_{r}\geq 1\;,\;r=1,\;\ldots ,\;s$$

$${\;\emptyset }_{i}\leq 1\;,\;i=1,\;\ldots .,m$$

A Decision-Making Unit (DMU) is considered efficient when the objective function is equal to one. Otherwise, the DMU is inefficient.

If the data are solely ratios of input to output data or vice versa, then DEA models cannot determine the efficiency and patterns of the units. Therefore, this research will employ Ratio-based Data Envelopment Analysis (DEA-R) under conditions of data uncertainty using robust optimization.

**CCR– Ratio**


Min ∅

$$s_{.}t\;\left\{ \begin{array}{c} \sum_{j=1}^{n}j\left(\frac{\frac{x_{ij}\;}{y_{rj}}}{\frac{x_{io}}{y_{ro}}}\right)\leq \emptyset \;\;,\;i=1,\;\ldots .,m\;,\;r=1,\;\ldots .,s\;\\ \;\\ \; \end{array}\right.\;$$ (11)

$$\sum_{j=1}^{n}j\geq 1\;$$

$${\mathrm{\backslash [}}_{j}\geq 0\;,\;j=1,\;\ldots .,n$$

A Decision-Making Unit (DMU) is considered efficient when the objective function ∅ is equal to one. Otherwise, the DMU is inefficient.

Notes concerning the CCR–Ratio model:

- The efficiency and super-efficiency obtained from this model are greater than or equal to the CCR model.
- The efficiency levels of this model are not necessarily equal when input-oriented and output-oriented.

**Additive–Ratio**


$$Max\;\sum_{i=1}^{m}\sum_{r=1}^{s}{\;s}_{ir}$$

$$s_{.}t\;\left\{ \begin{array}{c} \sum_{j=1}^{n}j\left(\frac{\frac{x_{ij}\;}{y_{rj}}}{\frac{x_{io}}{y_{ro}}}\right)+\;s_{ir}=1\;\;\\ \;\\ \; \end{array}\right.\;i=1,\;\ldots .m\;,\;r=1,\;\ldots .,s$$ (12)

$$\;\sum_{j=1}^{n}j\geq 1\;$$

$${\mathrm{\backslash [}}_{j}\geq 0\;,\;j=1,\;\ldots .,n\;$$

$$s_{ir}\geq 0\;,\;i=1,\;\ldots .,m\;,\;r=1,\;\ldots ,\;s$$

A Decision-Making Unit (DMU) is considered efficient when the objective function is equal to zero. Otherwise, the DMU is inefficient.

**Russell–Ratio**


$$min\;\frac{1}{mxs}\;\sum_{i=1}^{m}\;\sum_{r=1}^{s}\emptyset_{ir}$$ (13)

$$s_{.}t\;\left\{ \begin{array}{c} \sum_{j=1}^{n}j\left(\frac{\frac{x_{ij}\;}{y_{rj}}}{\frac{x_{io}}{y_{ro}}}\right)\leq \;\emptyset_{ir}\;\;\\ \;\\ \; \end{array}\right.\;i=1,\;\ldots .,m\;,\;r=1,\;\ldots .,s$$

$$\sum_{j=1}^{n}j\geq 1\;$$

$${\mathrm{\backslash [}}_{j}\geq 0\;,\;j=1,\;\ldots .,n\;$$

$$\emptyset_{ir}\leq 1\;,\;i=1,\;\ldots .,m\;,\;r=1,\;\ldots ,\;s$$

A Decision-Making Unit (DMU) is considered efficient when the objective function is equal to one. Otherwise, the DMU is inefficient. Mozafari et al [64]

### 3.3. Robust optimization

The first research conducted on robust optimization was introduced by Soyster [65] in the early 70s as a linear optimization model. This model gives us the best feasible solution to all the input data so that each input data can take any value in a range. This approach tends to find highly conservative solutions, meaning we significantly move away from the optimality of the nominal problem to ensure solution robustness. Soyster’s method is highly protective, extremely conservative in practice, and much worse than the optimal solution of the nominal problem in the sensitivity analysis on the robust solution of the objective function.

Ben–Tal & Nemirovski [66,67] proposed their robust optimization model based on ellipsoidal sets, which can control conservatism to resolve the issue of Soyster’s model.

Since the Ben–Tal & Nemirovski model is a nonlinear second-order cone problem, it cannot be used for discrete optimization problems as it increases the complexity of the problem; this is because a linear model with the Ben–Tal & Nemirovski approach changes into a nonlinear model.

The Bertsimas & Sim approach employs interval uncertainty sets and remains a linear model, making it the most widely used robustification approach. During the robustification of integer models with the Ben–Tal & Nemirovski approach, it is possible to solve them quickly in polynomial time. This model can adjust the level of conservatism, which only happens through the adjustment of one parameter. The symbol indicates the number of parameters that can have their maximum value simultaneously.

**Soyster**


$$\left\{ \begin{array}{c} \sum b\;a_{ab}\phi_{b}+\;\sum b\in \Lambda_{a}\;{\hat{\alpha }}_{ab\;\Phi_{b}\leq \beta_{a},\;\forall_{a}}\\ -\Phi_{b}\leq \phi_{b}\leq \Phi_{b}.\;\forall b\;\\ \Phi \geq \;0\; \end{array}\right.\;$$ (14)

**Ben – Tal & Nemirovski**


$$\sum b\;\;{\hat{\alpha }}_{ab}\phi_{b}\leq \beta_{a},\;\forall_{a}\;\left\{ \begin{array}{c} \sum b\;a_{ab}\phi_{b}+\;\sum b\in \Lambda_{a}\;{\hat{\alpha }}_{ab}\Phi ab\;{+}_{a}\sqrt{\sum b\in \Lambda_{a}\;{\hat{\alpha }}_{ab}^{2}}\sigma_{ab}^{2}\leq \beta_{a},\;\forall a\\ -\Phi_{ab}\leq \phi_{b}-\sigma_{ab}\leq \;\Phi_{ab},\forall a,\;b\in_{a}\;\\ \Phi \geq \;0\; \end{array}\right.\;$$ (15)

**Bertsimas & Sim [27]**


$$\left\{ \begin{array}{c} \sum_{b}a_{ab}\phi_{b}+z_{a}\Gamma_{a}+\sum_{b\in \Lambda_{a}}p_{ab}\leq \beta_{a},\;\forall_{a}\\ z_{a}+p_{ab}\geq {\hat{\alpha }}_{ab}\;\Phi_{b},\forall a,\;b\in \Lambda_{a}\\ -\Phi_{b}\leq \phi_{b}\leq \Phi_{b},\forall a,\;b\in \Lambda_{a} \end{array}\right.\;$$ (16)

z,p,Φ≥ 0

### 3.4. Robust ratio-based data envelopment analysis models under data uncertainty

In this research, the three RR-DEA new models proposed below are applied in scenarios where the intended data are expressed as ratios between inputs and outputs or vice versa. There is also uncertainty regarding these data ratios.

Bertsimas and Sim method (Uncertainty is considered as an interval). A linear model with a level of conservatism can be controlled and the model remains linear, hence it is the most widely used robust approach. When establishing integer models with this approach, it is possible to quickly solve them in the time of a polynomial. This model has the ability to adjust the conservatism level. Therefore, this method has been used to make robust DEA models in ratio conditions.

**(RR-CCR)**


Min ∅

$$s_{.}t\;\left\{ \begin{array}{c} \sum_{j=1}^{n}{(}_{j}\left(\frac{\frac{x_{ij}\;}{y_{rj}}}{\frac{x_{io}}{y_{ro}}}\right)+\;z_{j}\Gamma_{j}+p_{j})\leq \emptyset \;,\;\forall_{i,r}\;\;\\ \;\\ \; \end{array}\right.\;$$ (17)

$$\sum_{j=1}^{n}{(}_{j}+\;z_{j}\Gamma_{j}+p_{j})\geq 1\;$$

$${\;z_{j}+p_{j}\;\geq \Delta \;.\;}_{j}.\left(\frac{\frac{x_{ij}\;}{y_{rj}}}{\frac{x_{io}}{y_{ro}}}\right)\;,\;\forall_{i,r,j}\;$$

$${\;z_{j}\;,\;p_{j}\;,\;}_{j}\geq 0\;,\;\forall_{j}\;$$

**(RR– Additive)**


$$Max\;\sum_{i=1}^{m}\;\sum_{r=1}^{s}s_{ir}$$

$$s_{.}t\;\left\{ \begin{array}{c} \sum_{j=1}^{n}{(}_{j}\left(\frac{\frac{x_{ij}\;}{y_{rj}}}{\frac{x_{io}}{y_{ro}}}\right)+\;z_{j}\Gamma_{j}+p_{j})+\;s_{ir}=1\;,\forall_{i,r}\;\;\\ \;\\ \; \end{array}\right.\;$$ (18)

$$\sum_{j=1}^{n}{(\lambda }_{j}+\;z_{j}\Gamma_{j}+p_{j})\geq 1\;$$

$${\;z_{j}+p_{j}\;\geq \Delta \;.\;}_{j}.\left(\frac{\frac{x_{ij}\;}{y_{rj}}}{\frac{x_{io}}{y_{ro}}}\right)\;,\;\forall i,r,j\;$$

$${z_{j}\;,\;p_{j}\;,\;}_{j},\;s_{ir}\geq 0\;,\;\forall_{i,r,j}$$

**(RR – Russell)**


$$Min\frac{1}{mxs}\;\sum_{i=1}^{m}\;\sum_{r=1}^{s}\emptyset_{ir}$$

$$s_{.}t\;\left\{ \begin{array}{c} \sum_{j=1}^{n}{(\lambda }_{j}\left(\frac{\frac{x_{ij}\;}{y_{rj}}}{\frac{x_{io}}{y_{ro}}}\right)+\;z_{j}\Gamma_{j}+p_{j})\leq \;\emptyset_{ir}\;,\forall_{i,r}\;\;\\ \;\\ \; \end{array}\right.\;$$ (19)

$$\sum_{j=1}^{n}{(\lambda }_{j}+\;z_{j}\Gamma_{j}+p_{j})\geq 1\;$$

$${\;z_{j}+p_{j}\;\geq \Delta \;.\;}_{j}.\left(\frac{\frac{x_{ij}\;}{y_{rj}}}{\frac{x_{io}}{y_{ro}}}\right)\;,\;\forall i,r,j\;$$

$${z_{j}\;,\;p_{j}\;,\;}_{j}\geq 0\;,\;\forall_{j}$$

$$\emptyset_{ir}\leq 1\;,\;i=1,\;\ldots .,m\;,\;r=1,\;\ldots ,\;s$$

### 3.5. The proposed ANFIS algorithm to forecast stock prices

In the second stage of modeling, the enhanced Adaptive Neuro-Fuzzy Inference System (ANFIS) approach is employed to forecast stock prices with high precision, as follows:

**ANFIS Algorith** **m**

% Algorithm for time series prediction

% loading Tehran Stock series data

% total points 1200 in this series

load TSdata.mat

% input data

time = TSdata(:,1);

% output data

x = TSdata(:,2);

figure(1)

plot(time,x) %TS series plot

title('Tehran-Stock chaotic time series')

xlabel('time')

ylabel('x(t)')

% preparing input output data matrix

% first four columns of data, are input for ANFIS and last column is output

for t = 118:1117

 data(t-117,:) = [x(t-3) x(t-2) x(t-1) x(t) x(t + 1)];

end

% first 500 points for training

trndata = data(1:500,:);

% last points for validation

valdata = data(501:end,:);

% generating fis

fis = genfis3(trndata(:,1:end-1),trndata(:,end),'sugeno',7);

% defining training options for ANFIS

options = anfisOptions('StepSizeDecreaseRate',0.9,'InitialStepSize',0.01,'InitialFIS',fis,'ErrorGoal',0.001,'EpochNumber',50,'ValidationData',valdata);

% training ANFIS

[fis1,error1,ss,fis2,error2] = anfis(trndata,options);

% Evaluating ANFIS for 6 samples prediction

anfis_output = evalfis(fis2,[trndata(:,1:4); valdata(:,1:4)]);

% plotting input TS series and predicted series

index = 124:1123;

% six future samples

index1 = 1118:1123;

figure(2)

plot(time(index),[x(index) anfis_output])

xlabel('Time')

title('TS series and ANFIS prediction')

figure(3)

plot(time(index1 + 1),[x(index1) anfis_output(end-5:end)])

hold on;

plot(time(index1 + 1),[x(index1) anfis_output(end-5:end)],'o','MarkerFaceColor','K')

xticks(index1)

xlabel('Time')

title('Next 6 samples prediction and actual TS series')

% Plotting Training and Validation error curves

figure(4)

plot([error1 error2]); hold on; plot([error1 error2],'o')

legend('Training error','Validation error')

xlabel('Epochs'); ylabel('RMS Error')

title('Error Plots')

% Finding Error

diff = x(index) – anfis_output;

figure(5)

plot(time(index),diff)

xlabel('Time'); title('Prediction Errors')

title('Difference in prediction')

Adaptive fuzzy neural inference system (ANFIS) combines the advantages of artificial neural networks (ANN) and fuzzy logic in a unit framework. It provides rapid learning capacity and adaptive interpretation capabilities for modeling complex patterns and understanding nonlinear relationships. (ANFIS) is the most popular fuzzy neural model for approximating very complex and nonlinear systems. ANFIS is generally very efficient as long as the number of inputs is less than five. Considering that we have only considered the close price as an input for predicting stock prices, we used this method over other prediction and machine learning methods.

### 3.6. Developing a fuzzy goal programming model for stock portfolio optimization

**Fig 3:**
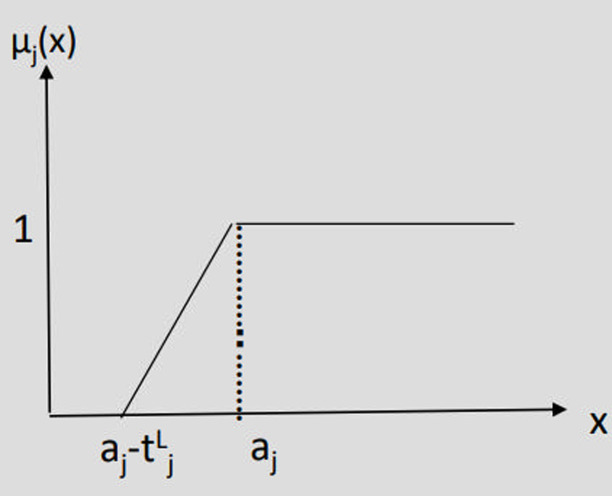
Maximizing fuzzy goal.

**Fig 4:**
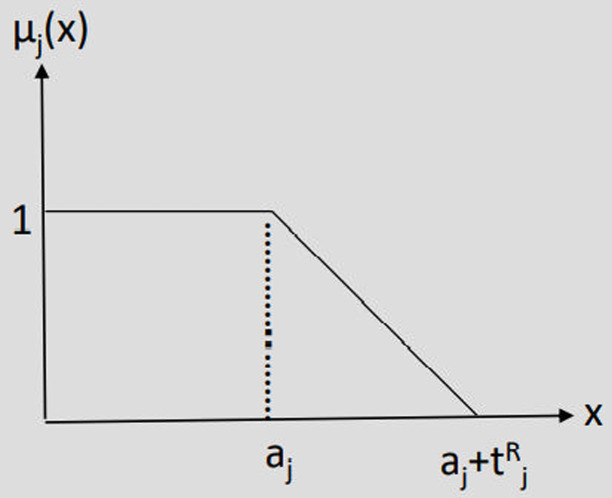
Minimizing fuzzy goal.

**Fig 5:**
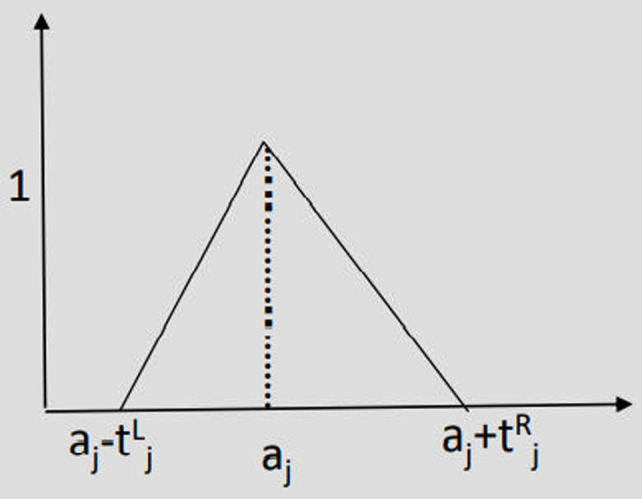
Any adverse deviation in fuzzy goal.


One of the challenges in the goal programming of deterministic problems is the precise determination of target values (threshold levels), denoted as b_i_ which is often impractical. Fuzzy theory offers a solution by allowing these values to be expressed imprecisely, leading to the formation of fuzzy goals. In the following fuzzy relations, a_j_ represents the goal value. Maximizing fuzzy goal, Minimizing fuzzy goal and Any adverse deviation in fuzzy goal are presented in Figs 3–5.

$${FIND}_{X}$$

s.t.

$$F_{j}(X)\;\tilde{>}\;a_{j\;}\to \;Maximization$$

$$F_{j}(X)\;\tilde{<}\;a_{j\;}\;\to \;Minimization\;$$

$$F_{j}(X)\;≅\;a_{j\;}\;\to \;Any\;Adverse\;Deviation$$

ax≤b

Maximization


$$F_{j}(X)\;\tilde{>}\;a_{j\;}$$

$$\mu_{j}(X)=\;\left\{ \begin{array}{c} \;1\;{\;f}_{j}\;(X)\geq \;a_{j\;}\;\\ \;\\ \frac{f_{j}\;(X)-(a_{j}-\;t_{j}^{l})\;}{t_{j}^{l}\;}\;a_{j}-\;t_{j}^{l}<\;f_{j}\;(X)<\;a_{j}\;\\ \;\\ \;0\;f_{j}\;(X)\;\leq \;a_{j}-\;t_{j}^{l} \end{array}\right.\;$$ (20)

Minimization


$$F_{j}(X)\;\tilde{<}\;a_{j\;}$$

$$\mu_{j}(X)=\;\left\{ \begin{array}{c} \;1\;{\;f}_{j}\;(X)\leq \;a_{j\;}\;\\ \;\\ \frac{a_{j}+\;t_{\;j}^{R}-f_{j}\;(X)\;}{t_{\;j\;}^{R}\;}\;a_{j}<\;f_{j}\;(X)<\;a_{j}+\;t_{\;j}^{R}\;\\ \;\\ \;0\;f_{j}\;(X)\;\geq \;a_{j}+\;t_{\;j}^{R} \end{array}\right.\;$$ (21)

Any Adverse Deviation

**Fuzzy Ideal**


$$F_{j}(X)\;≅\;a_{j\;}$$

$$\mu_{j}(X)=\;\left\{ \begin{array}{c} \;\frac{f_{j}\;(X)-(a_{j}-\;t_{\;j}^{L})\;}{t_{\;j\;}^{L}\;}\;a_{j}-\;t_{\;j}^{L}\;\leq {\;f}_{j}\;(X)\leq \;a_{j\;}\;\\ \;\\ \frac{a_{j}+\;t_{\;j}^{R}-f_{j}\;(X)\;}{t_{\;j\;}^{R}\;}\;a_{j}\leq \;f_{j}\;(X)\leq \;a_{j}+\;t_{\;j}^{R}\;\\ \;\\ \;0\;Otherwise\; \end{array}\right.\;$$ (22)

**Fuzzy goal programming model**

Min Z = D


$$s_{.}t\;\left\{ \begin{array}{c} \frac{1}{k_{i}}\;\left(u_{i}n_{i}+\;v_{i}p_{i}\right)\leq D\;,\;\forall_{i}\;\\ \;\sum_{j=1}^{n}a_{ij}x_{j}\;+\;n_{i}-\;p_{i}=\;{\tilde{b}}_{i}\;,\forall_{i}\;\;\\ \;\\ \; \end{array}\right.\;$$ (23)

$$\sum_{j=1}^{n}x_{j}=1\;,\;0\leq x_{j}\leq 1\;j=1,\ldots ,n\;$$

$$all\;n_{i\;},\;p_{i}\;variables\;\geq 0\;,\forall_{i}$$

### 3.7. Fuzzy optimization using the Zimmerman method

The basis of all multi-objective decision-making methods in a fuzzy environment is the creation of a balance table and the creation of fuzzy membership functions for the objective functions. Zimmerman developed this approach in 1978 [68]. Balance table and the creation of fuzzy membership functions for the objective functions are presented in Table 1.

**Table 1:** Balance table and the creation of fuzzy membership functions for the objective functions.

$X_{\;i}^{*}$	Goal Direction	$Z_{1}$	$Z_{2}$	$Z_{3}$
$X_{\;1}^{*}$	$MinZ_{1}$	$Z_{\;1}^{\;PIS}$	$Z_{2}(X_{\;1}^{*})$	$Z_{3}(X_{\;1}^{*})$
$X_{\;2}^{*}$	$MaxZ_{2}$	$Z_{1}(X_{\;2}^{*})$	$Z_{\;2}^{\;PIS}$	$Z_{3}\;(X_{\;2}^{*})$
$X_{\;3}^{*}$	$MaxZ_{3}$	$Z_{1}(X_{\;3}^{*})$	$Z_{2}(X_{\;3}^{*})$	$Z_{\;3}^{\;PIS}$
$Z_{\;i}^{NIS}$		$Max\left\{Z_{1}\left(X_{\;2}^{*}\right),Z_{1}(X_{\;3}^{*})\right\}$	$Min\left\{Z_{2}\left(X_{\;1}^{*}\right),Z_{2}(X_{\;3}^{*})\right\}$	$Min\left\{Z_{3}\left(X_{\;1}^{*}\right),Z_{3}(X_{\;2}^{*})\right\}$


$$MinZ_{1}\;=\sum_{j=1}^{n}a_{1j}x_{j}$$ (24)

$$MaxZ_{2}\;=\sum_{j=1}^{n}a_{2j}x_{j}$$ (25)

$$MaxZ_{3}\;=\sum_{j=1}^{n}a_{3j}x_{j}$$ (26)

Step 1: Defining the ideal and anti-ideal solutions for the objective functions by solving three single-objective models.

Step 2: Establishing the membership function for each objective function based on the range of values obtained from the balance table


$$\mu_{z1}(X)=\;\left\{ \begin{array}{cc} 1 & Z_{1}(X)\leq \;Z_{1}^{\;PIS}\\ \frac{Z_{1}^{\;NIS}-X}{Z_{1}^{\;NIS}\;-Z_{1}^{\;PIS}\;} & Z_{1}^{\;PIS}\;<Z_{1}(X)<\;Z_{1}^{\;NIS}\\ 0 & Z_{1}(X)\geq \;Z_{1}^{\;NIS} \end{array}\right.\;$$ (27)

$$\mu_{z2}(X)=\;\left\{ \begin{array}{cc} 1 & Z_{2}(X)\geq \;Z_{2}^{\;PIS}\\ \frac{X-Z_{2}^{\;NIS}}{Z_{2}^{\;PIS}\;-Z_{2}^{\;NIS}\;} & Z_{2}^{\;NIS}\;<Z_{2}(X)<Z_{2}^{\;PIS}\\ 0 & Z_{2}(X)\leq \;Z_{2}^{\;NIS} \end{array}\right.\;$$ (28)

$$\mu_{z3}(X)=\;\left\{ \begin{array}{cc} 1 & Z_{3}(X)\geq \;Z_{3}^{\;PIS}\\ \frac{X-Z_{3}^{\;NIS}\;}{Z_{3}^{\;PIS}-Z_{3}^{\;NIS}\;} & Z_{3}^{\;NIS}\;<Z_{3}(X)<\;Z_{3}^{\;PIS}\\ 0 & Z_{3}(X)\leq \;Z_{3}^{\;NIS} \end{array}\right.\;$$ (29)

Step 3: Converting the initial multi-objective model into an equivalent single-objective model using an aggregation function.

**Zimmerman Model**

$\;=\min\left\{\mu_{zi}(X)\right\}$ λ: The minimum satisfaction level of the objective function


Max (30)

s.t.

$$\mu_{zi}(X)\geq$$

X∈S

∈[0, 1]

Max (31)

s.t.

$$X+\;\left(Z_{1}^{\;NIS}-\;Z_{1}^{\;PIS}\right)\leq Z_{1}^{\;NIS}$$

$$X-\;\left(Z_{2}^{\;PIS}-\;Z_{2}^{\;NIS}\right)\geq Z_{2}^{\;NIS}$$

$$X-\;\left(Z_{3}^{\;PIS}-\;Z_{3}^{\;NIS}\right)\geq Z_{3}^{\;NIS}$$

$$X\in F_{X}$$

 ∈[0, 1]

### 3.8. Testing and implementing the proposed models

GAMS and MATLAB will be used to implement the proposed models. Thus, several data related to selected stocks, based on particular indices, will be selected from the Tehran Stock Exchange (TSE) market in specific periods and utilized in the software to test the proposed models.

## 4. Data analysis

### 4.1. Introduction

**Fig 6:**
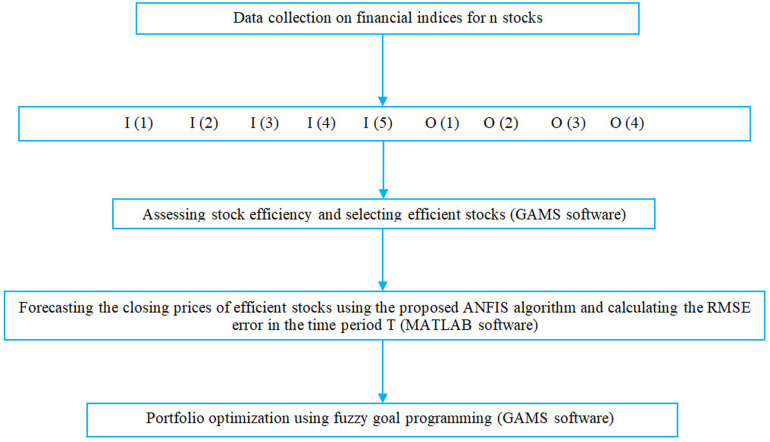
Research flowchart.

This section will first focus on the analyses, inputs, and results by identifying and introducing the existing data and also testing the proposed RR-DEA models. Then, efficient stocks will be chosen for the first stage of modeling. In the second stage, using the proposed ANFIS model, the prices of efficient stocks are forecast within the period T, and RMSE will be employed as the prediction error measure. Last, the final stage will employ multi-objective decision-making (MODM) methods for portfolio optimization, and the amount of each stock to be purchased will be determined using the goal programming method. Research flowchart is presented in Fig 6.

### 4.2. Data selection and introduction

Information on 27 existing stocks in the pharmaceutical industry will be used as a real-world sample from the Tehran Stock Exchange (TSE) with their financial indices data extracted from March 2013 to March 2014 to evaluate the proposed models. However, more data can be selected and optimized from stocks of other industries. Further, Table 2 depicts the selected variables of the Robust Ratio-based Data Envelopment Analysis (RR-DEA) models, along with the inputs and outputs of the models. Description of inputs and outputs of RR-DEA models are presented in Table 3.

**Table 2:** The selected variables of the robust ratio-based model.

Input Variables	Symbol	Output Variables	Symbol
The price-to-earnings (P/E) ratio per share	I(1)	Earnings per share (EPS)	O(1)
Quick ratio	I(2)	One-year returns	O(2)
Debt-to-equity ratio	I(3)	Liquidity ratio	O(3)
The Beta (β) Index is based on the industry’s returns.	I(4)	Earnings per share (EPS) growth rate	O(4)
The sigma index	I(5)		

**Table 3:** Description of inputs and outputs of RR-DEA models.

Symbol	Description
I(1)	Stock price divided by net income per share
I(2)	Total current assets minus inventory divided by total current liabilities
I(3)	Total liability divided by shareholders equity
I(4)	Systematic Risk
I(5)	Non-Systematic Risk
O(1)	Net income minus dividends divided by common shares
O(2)	Proportion of gain or loss on an investment over a specified period
O(3)	Degree which presents stock ability to be bought or sold in the market quickly
O(4)	Current quarters EPS divided by the previous quarters EPS minus one

The lower the DMU input and the higher the DMU output, the better. For this purpose, according to the following table, inputs and outputs have been selected and described.

Table 4 illustrates the information on existing stocks in the pharmaceutical industry.

**Table 4:** Information on the existing stocks of the pharmaceutical industry.

Symbol	DMU	I(1)	I(2)	I(3)	I(4)	I(5)	O(1)	O(2)	O(3)	O(4)
**PDRO**	DMU01	7.43	1.18	1.22	1.03	0.02	3344	1.93	157.67	59.33
**DLGM**	DMU02	13.38	0.49	3.87	0.7	0.03	213	2.06	183.48	133.33
**THSH**	DMU03	11.58	0.59	2.85	0.01	0.02	799	0.69	110.28	30.16
**DDPK**	DMU04	7.7	0.86	2.27	0.54	0.05	693	2.73	122.76	56.85
**TMVD**	DMU05	6.58	1.16	1	0.64	0.02	2965	1.04	166.99	10.66
**DAML**	DMU06	8.7	0.87	3.91	0.57	0.03	1386	1.98	156.08	2.74
**DFRB**	DMU07	7.76	1.07	1.84	1.4	0.03	1277	2.04	164.07	31.17
**DKSR**	DMU08	8.96	0.97	1.36	1.48	0.03	121	2.64	228.88	369.42
**DARO**	DMU09	7.93	7.07	0.1	1.27	0.03	1553	1.85	187.63	54.67
**DABO**	DMU10	9.03	0.86	3.44	0.71	0.03	1357	2.3	143.68	93.15
**DRZK**	DMU11	7.91	0.96	1.72	0.68	0.03	1493	2.88	167.43	96.65
**DOSE**	DMU12	18.43	1.06	1.23	1.56	0.04	997	1.92	169.7	67
**PKSH**	DMU13	6.41	0.9	5.95	1.67	0.03	528	0.73	227.86	53.22
**IRDR**	DMU14	7.47	0.72	3	1.09	0.03	306	1.59	187.99	230.39
**DALZ**	DMU15	7.46	1.28	1.21	1.49	0.03	956	2.49	205.22	111.3
**DSBH**	DMU16	8.39	1.35	0.86	1.6	0.04	2340	2.91	155.82	95.56
**DPAK**	DMU17	6.82	0.79	4.43	1.3	0.05	666	2.52	177.08	119.82
**DJBR**	DMU18	6.94	1.21	0.94	0.94	0.03	659	3.14	219.36	122.76
**KIMI**	DMU19	6.81	0.73	2.28	6.24	0.21	227	5.74	147.27	438.33
**EXIR**	DMU20	8.2	0.82	5.16	1.14	0.03	1283	3.14	198.36	118.24
**DSIN**	DMU21	7.52	1.21	0.84	0.97	0.03	1222	1.8	174.39	94.68
**ROZD**	DMU22	8.84	1.01	0.95	0.28	0.07	131	1.46	26.37	286.26
**AMIN**	DMU23	5.73	0.97	1.45	0.74	0.04	696	4.15	163.71	230.03
**DZAH**	DMU24	5.4	0.95	2.83	1.2	0.07	2699	2.35	44.51	129.27
**ABDI**	DMU25	10.22	0.6	4.81	0.59	0.03	404	2.21	181.41	83.42
**ALBZ**	DMU26	6.9	1	1.93	1.41	0.03	418	1.49	228.42	104.07
**DSOB**	DMU27	6.75	1.06	1.57	1.46	0.03	655	2.65	221.73	104.58

### 4.3. Result analysis on the data envelopment analysis models

After data collection, we enter the Robust Ratio-based Data Envelopment Analysis (DEA) models into GAMS and run them. By considering uncertainty for the ratio-based data in the DEA models for all the pharmaceutical stocks, we will set the ϖ and Δ parameters based on Tables 5–7. We will then run the models. The tables below show the results of the RR-CCR, RR-Additive, and RR-Russell models.

**Table 5:** Results for the RR-CCR model.

Stock	Γ = 0.25		Γ = 0.5		Γ = 1	
	∆=0.01	∆ =0.1	∆ =0.01	∆ =0.1	∆ =0.01	∆ =0.1
**PDRO**	1	1	1	1	1	1
**DLGM**	1	1	1	1	1	1
**THSH**	1	1	1	1	1	1
**DDPK**	0.9762	0.9770	0.9763	0.9778	0.9765	0.9793
**TMVD**	1	1	1	1	1	1
**DAML**	1	1	1	1	1	1
**DFRB**	0.9064	0.9096	0.9068	0.9130	0.9075	0.9188
**DKSR**	1	1	1	1	1	1
**DARO**	1	1	1	1	1	1
**DABO**	1	1	1	1	1	1
**DRZK**	1	1	1	1	1	1
**DOSE**	0.9681	0.9692	0.9682	0.9705	0.9685	0.9726
**PKSH**	1	1	1	1	1	1
**IRDR**	1	1	1	1	1	1
**DALZ**	1	1	1	1	1	1
**DSBH**	1	1	1	1	1	1
**DPAK**	0.9983	0.9983	0.9983	0.9984	0.9983	0.9985
**DJBR**	1	1	1	1	1	1
**KIMI**	1	1	1	1	1	1
**EXIR**	1	1	1	1	1	1
**DSIN**	1	1	1	1	1	1
**ROZD**	1	1	1	1	1	1
**AMIN**	1	1	1	1	1	1
**DZAH**	1	1	1	1	1	1
**ABDI**	1	1	1	1	1	1
**ALBZ**	1	1	1	1	1	1
**DSOB**	1	1	1	1	1	1

**Table 6:** Results for the RR-Additive model.

Stock	Γ = 0.25		Γ = 0.5		Γ = 1	
	∆ =0.01	∆ =0.1	∆ =0.01	∆ =0.1	∆ =0.01	∆ =0.1
**PDRO**	0	0	0	0	0	0
**DLGM**	0	0	0	0	0	0
**THSH**	0	0	0	0	0	0
**DDPK**	6.8072	6.5966	6.7832	6.3774	6.7356	5.9800
**TMVD**	0	0	0	0	0	0
**DAML**	0	0	0	0	0	0
**DFRB**	7.5644	7.3864	7.5442	7.2096	7.5041	6.8822
**DKSR**	0	0	0	0	0	0
**DARO**	0	0	0	0	0	0
**DABO**	0	0	0	0	0	0
**DRZK**	0	0	0	0	0	0
**DOSE**	7.6747	7.4212	7.6456	7.1641	7.5883	6.7091
**PKSH**	0	0	0	0	0	0
**IRDR**	0	0	0	0	0	0
**DALZ**	0	0	0	0	0	0
**DSBH**	0	0	0	0	0	0
**DPAK**	3.8009	3.6819	3.7873	3.5582	3.7604	3.3341
**DJBR**	0	0	0	0	0	0
**KIMI**	0	0	0	0	0	0
**EXIR**	0	0	0	0	0	0
**DSIN**	0	0	0	0	0	0
**ROZD**	0	0	0	0	0	0
**AMIN**	0	0	0	0	0	0
**DZAH**	0	0	0	0	0	0
**ABDI**	0	0	0	0	0	0
**ALBZ**	0	0	0	0	0	0
**DSOB**	0	0	0	0	0	0

**Table 7:** Results for the RR-Russell model.

Stock	Γ = 0.25		Γ = 0.5		Γ = 1	
	∆ = 0.01	∆ = 0.1	∆ = 0.01	∆ = 0.1	∆ = 0.01	∆ = 0.1
**PDRO**	1	1	1	1	1	1
**DLGM**	1	1	1	1	1	1
**THSH**	1	1	1	1	1	1
**DDPK**	0.6596	0.6702	0.6608	0.6811	0.6632	0.7010
**TMVD**	1	1	1	1	1	1
**DAML**	1	1	1	1	1	1
**DFRB**	0.6218	0.6307	0.6228	0.6395	0.6248	0.6559
**DKSR**	1	1	1	1	1	1
**DARO**	1	1	1	1	1	1
**DABO**	1	1	1	1	1	1
**DRZK**	1	1	1	1	1	1
**DOSE**	0.6163	0.6289	0.6177	0.6418	0.6206	0.6645
**PKSH**	1	1	1	1	1	1
**IRDR**	1	1	1	1	1	1
**DALZ**	1	1	1	1	1	1
**DSBH**	1	1	1	1	1	1
**DPAK**	0.8100	0.8159	0.8106	0.8221	0.8120	0.8333
**DJBR**	1	1	1	1	1	1
**KIMI**	1	1	1	1	1	1
**EXIR**	1	1	1	1	1	1
**DSIN**	1	1	1	1	1	1
**ROZD**	1	1	1	1	1	1
**AMIN**	1	1	1	1	1	1
**DZAH**	1	1	1	1	1	1
**ABDI**	1	1	1	1	1	1
**ALBZ**	1	1	1	1	1	1
**DSOB**	1	1	1	1	1	1

As observed from the results of Tables 5–7, due to inefficiency, the stocks of DDPK, DFRB, DOSE and DPAK are omitted from the ways of RR-DEA in the first stage of optimization. Out of 27 selected stocks in stage 1 from ways of RR-DEA, 23 efficient stocks are chosen to enter the second stage to have their prices predicted. 23 stocks were selected from RR-DEA for the second stage.

### 4.4. Testing, validating, and analyzing the ANFIS algorithm results

**Fig 7:**
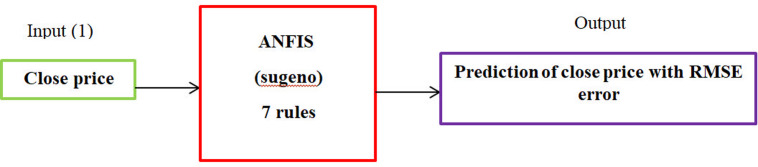
The ANFIS input and output.


In the second stage, the proposed ANFIS algorithm is employed to forecast the closing prices of 23 stocks selected from stage 1. Thus, data regarding closing prices from 02/03/2013–04/02/2018 are extracted from the Tehran Stock Exchange website as a time series for all the selected stocks. After data collection, we will normalize them using the standard normalization method. The ANFIS input and output are presented in Fig 7.

$$\frac{x-\mu }{\delta }$$ (32)

After coding the mentioned algorithm in MATLAB, we input the normalized data of every stock to forecast their prices and calculate the RMSE error in training and testing data sets. The ANFIS algorithm details are presented in Table 8. Table 9 illustrates their results.

**Table 8:** The ANFIS algorithm details.

ANFIS Details	
No. of nodes	77
No. of linear parameters	35
No. of nonlinear parameters	56
Total No. of parameters	91
No. of training data pairs	500
No. of assessed data pairs	500
No. of fuzzy rules	7
No. of repetition	50

**Table 9:** Forecast error results of the price of each stock.

Minimal training RMSE		Minimal testing RMSE	
Stock	RMSE	Stock	RMSE
PDRO	0.097	PDRO	0.051
DLGM	0.0997	DLGM	0.164
THSH	0.0152	THSH	0.0508
TMVD	0.069	TMVD	0.2396
DAML	0.139	DAML	0.044
DKSR	0.109	DKSR	0.0201
DARO	0.0716	DARO	0.104
DABO	0.105	DABO	0.206
DRZK	0.047	DRZK	0.240
PKSH	0.115	PKSH	0.099
IRDR	0.102	IRDR	0.062
DALZ	0.146	DALZ	0.144
DSBH	0.115	DSBH	0.106
DJBR	0.055	DJBR	0.069
KIMI	0.106	KIMI	0.062
EXIR	0.062	EXIR	0.441
DSIN	0.039	DSIN	0.275
ROZD	0.064	ROZD	0.099
AMIN	0.159	AMIN	0.014
DZAH	0.044	DZAH	0.127
ABDI	0.0201	ABDI	1.709
ALBZ	0.117	ALBZ	0.102
DSOB	0.118	DSOB	0.123

**Fig 8:**
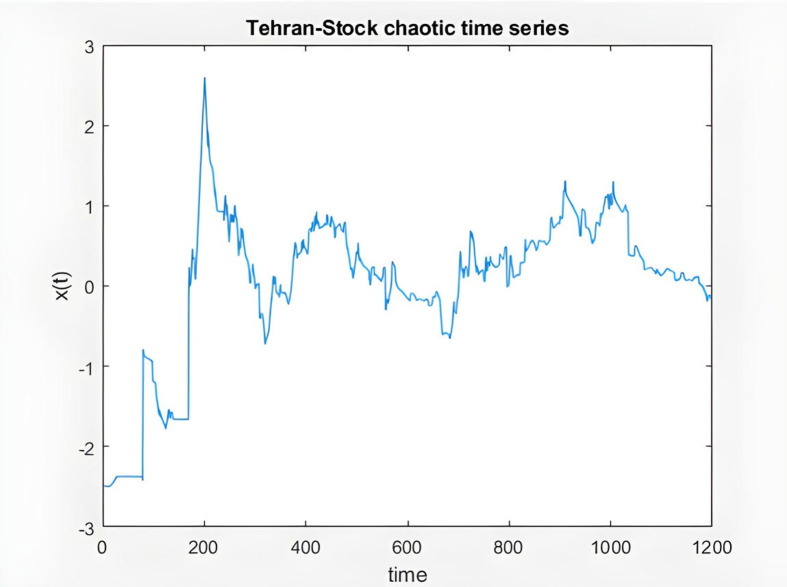
Chaotic time series from KIMI stock data.

**Fig 9:**
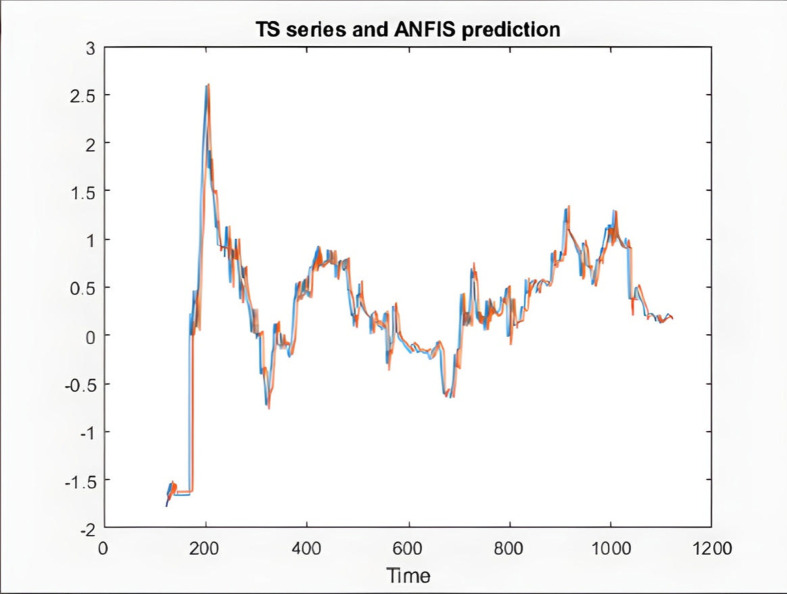
ANFIS prediction and the time series of KIMI stock.

**Fig 10:**
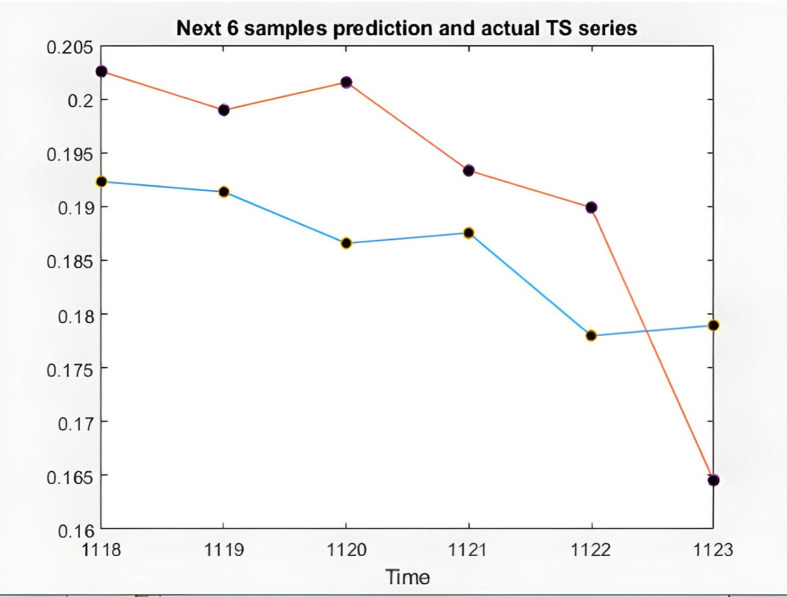
6 samples of ANFIS prediction and the actual time series of KIMI stock.

**Fig 11:**
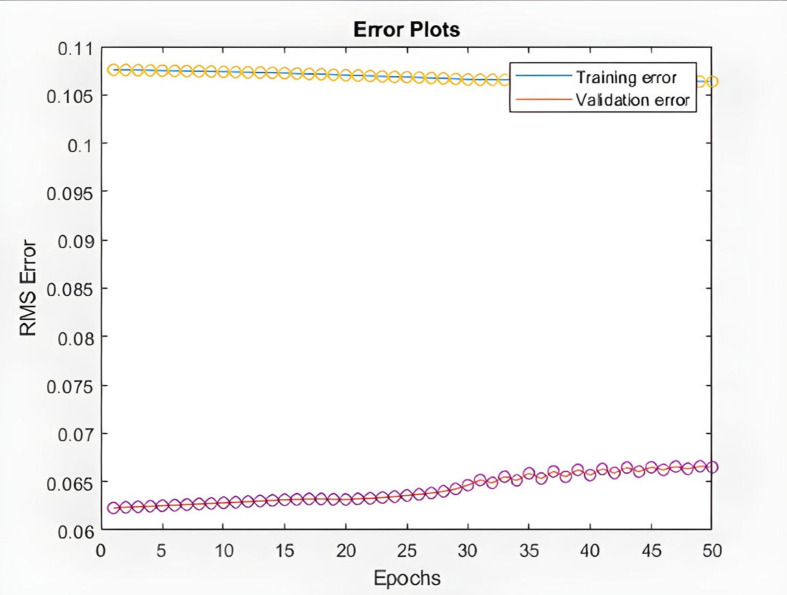
Validating and training error plots based on the number of repetitions (KIMI stock).

**Fig 12:**
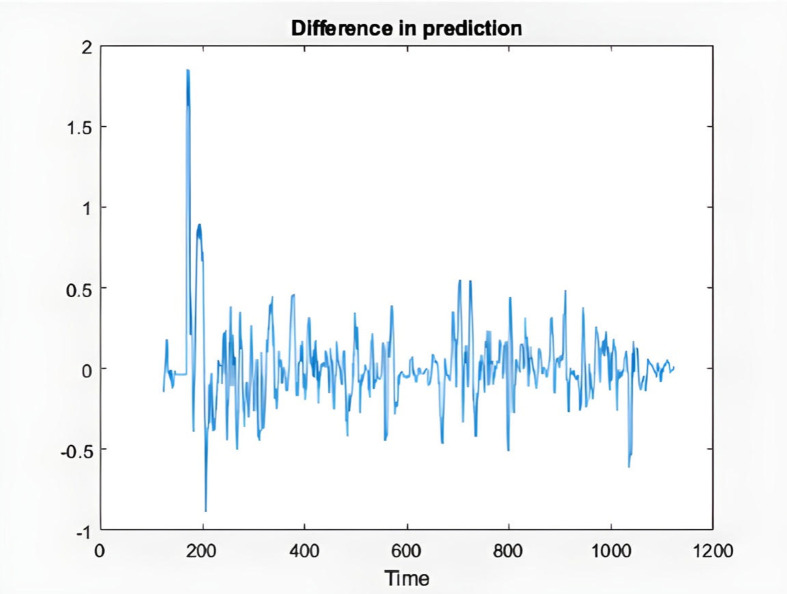
Difference in the prediction of KIMI stock.

**Fig 13:**
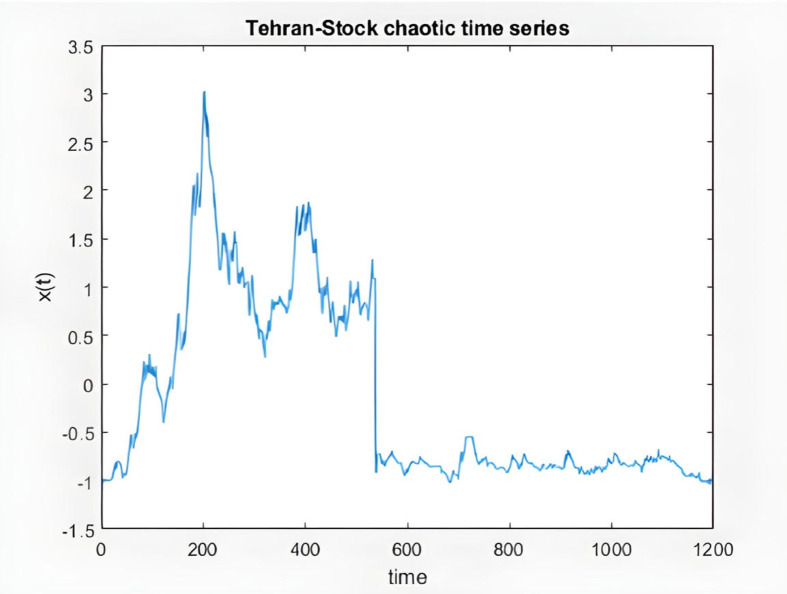
Chaotic time series from DKSR stock data.

**Fig 14:**
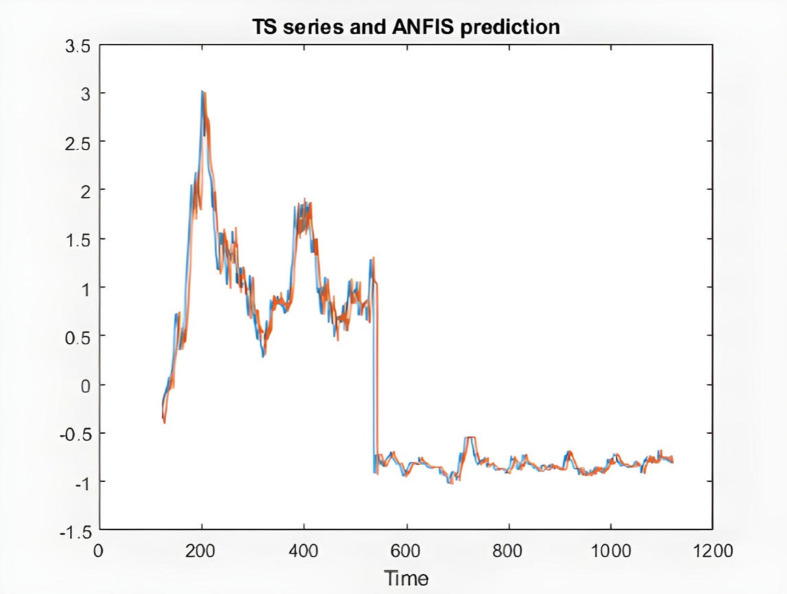
ANFIS prediction and the time series of DKSR stock.

**Fig 15:**
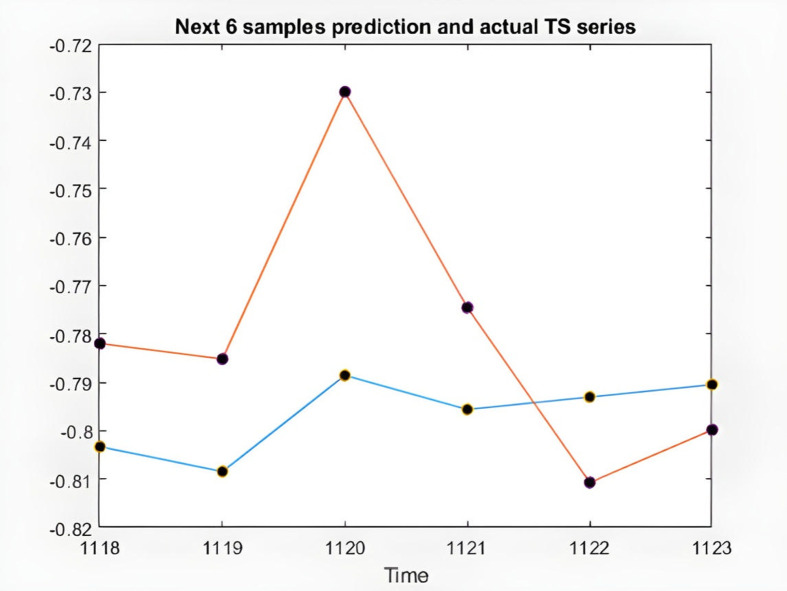
6 samples of ANFIS prediction and the actual time series of DKSR stock.

**Fig 16:**
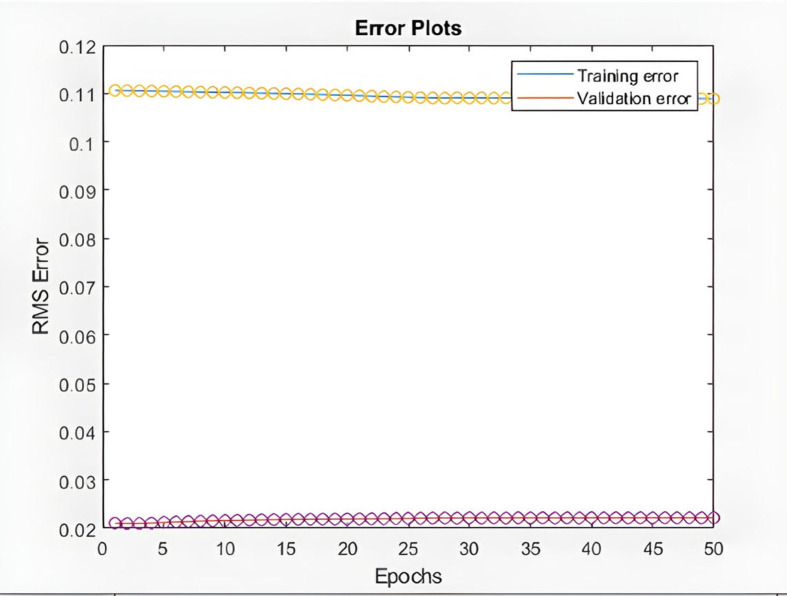
Validating and training error plots based on the number of repetitions (DKSR stock).

**Fig 17:**
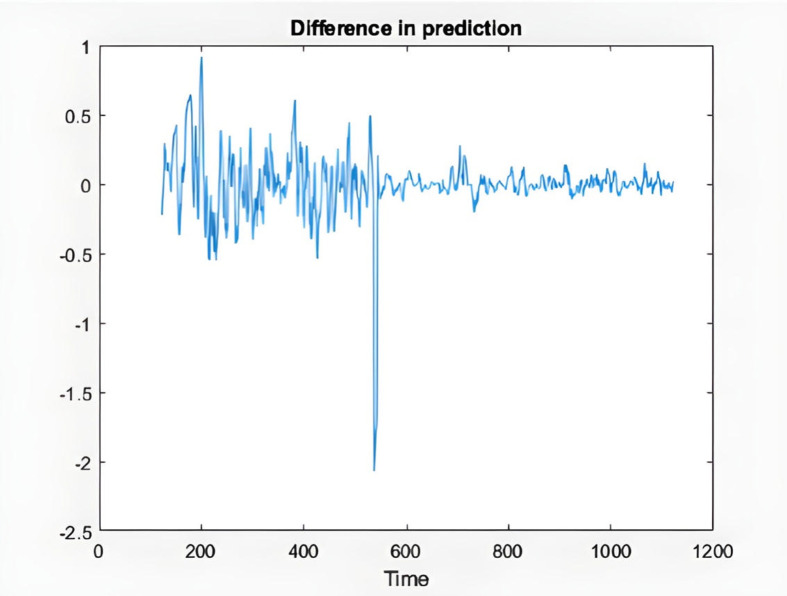
Difference in the prediction of DKSR stock.

In the following, the charts of KIMI and DKSR stocks are depicted as an example to analyze the ANFIS prediction results. The charts schematically show the time series of the chaotic stock data, the ANFIS prediction, and the stock time series, the next six samples of ANFIS prediction and the actual time series, validating and training error plots based on the number of repetitions, and finally the difference in prediction in Figs 8–17.

Artificial Neural Network (ANN), consists of an arbitrary number of neurons (processing units) that are placed in different layers that connect the input set to the output. Neurons are the basis of the structure of neural networks. A neuron takes a number of inputs, processes them, and then produces an output. When we design a network, our goal is to increase prediction accuracy and reduce prediction errors. When we want to train a neural network, instead of sending the entire input, we divide it into small packets of the same size. When the data is sent in batches, our model can be trained better than the model that received all the data at once. A round-trip propagation in the network is called an epoch, which is an arbitrary number. Also, if the number of epochs is too large, the network may be over-fit. Convolutional Neural Network (CNN) is a deep learning algorithm that is widely used in natural language processing, image processing and pattern recognition. The most important difference between the CNN network and other neural networks is the less need for data pre-processing and determining network characteristics. Meanwhile, in other methods the desired features are manually engineered and calculated. During learning, some neurons let go randomly. This means that learning takes place on different structures with different sets of neurons. In other words, in this technique, the output of several networks is mixed to form the final output. ANFIS is a fuzzy neural inference system, a type of artificial neural network. Since this system integrates neural networks and fuzzy logic concepts, it can take advantage of both of them in one system. Fuzzy inference system is according to the set of if-then rules, which uses the learning ability of the neural network and therefore has the ability to approximate non-linear functions. Therefore, ANFIS has been proposed as a universal approximation and has a better efficiency percentage.

### 4.5. Optimizing the stock portfolio using the fuzzy goal programming method

Fuzzy goal programming is an efficient tool for modeling real-life decision-making problems. Goal programming is an attempt to extend linear planning to cater to problems with multiple objectives. This approach requires lowering the ideal level for some objectives and reducing deviations from ideal levels. When objectives are unequal, the priority of each goal is captured by the deviation variables of the goal. In most real-world situations, the goals of the decision-maker, the priority factors, and even the importance assigned to goals are often vague. Fuzzy set theory addresses this issue. The key difference between traditional goal programming and fuzzy goal programming is the way the ideal values are defined. In goal programming, the decision-maker must specify a set of precise ideal values for each objective. In contrast, fuzzy goal programming allows these values to be defined imprecisely, accommodating the inherent uncertainty in real-world decision-making.

According to Table 10, it is clear that the information concerning the stocks that have been used in the optimization has been determined by using the goal programming method. However, to find the best solution for each objective function, some steps are needed, depending on whether the objective is to maximize or minimize. For the first six objective functions, which are of the minimization type, the ideal state of the Z_PIS_ is set to the lowest available value for each Index. On the other hand, for the next four objective functions, which are of the maximization type, the maximum value of each Index is considered the ideal state of Z_PIS_.

**Table 10:** Data regarding the stocks involved in goal programming optimization method.

Symbol	DMU	I(1)	I(2)	I(3)	I(4)	I(5)	I(6)	O(1)	O(2)	O(3)	O(4)
PDRO	DMU01	7.43	1.18	1.22	1.03	0.02	0.097	3344	1.93	157.67	59.33
DLGM	DMU02	13.38	0.49	3.87	0.7	0.03	0.0997	213	2.06	183.48	133.33
THSH	DMU03	11.58	0.59	2.85	0.01	0.02	0.0152	799	0.69	110.28	30.16
TMVD	DMU04	6.58	1.16	1	0.64	0.02	0.069	2965	1.04	166.99	10.66
DAML	DMU05	8.7	0.87	3.91	0.57	0.03	0.139	1386	1.98	156.08	2.74
DKSR	DMU06	8.96	0.97	1.36	1.48	0.03	0.109	121	2.64	228.88	369.42
DARO	DMU07	7.93	7.07	0.1	1.27	0.03	0.0716	1553	1.85	187.63	54.67
DABO	DMU08	9.03	0.86	3.44	0.71	0.03	0.105	1357	2.3	143.68	93.15
DRZK	DMU09	7.91	0.96	1.72	0.68	0.03	0.047	1493	2.88	167.43	96.65
PKSH	DMU10	6.41	0.9	5.95	1.67	0.03	0.115	528	0.73	227.86	53.22
IRDR	DMU11	7.47	0.72	3	1.09	0.03	0.102	306	1.59	187.99	230.39
DALZ	DMU12	7.46	1.28	1.21	1.49	0.03	0.146	956	2.49	205.22	111.3
DSBH	DMU13	8.39	1.35	0.86	1.6	0.04	0.115	2340	2.91	155.82	95.56
DJBR	DMU14	6.94	1.21	0.94	0.94	0.03	0.055	659	3.14	219.36	122.76
KIMI	DMU15	6.81	0.73	2.28	6.24	0.21	0.106	227	5.74	147.27	438.33
EXIR	DMU16	8.2	0.82	5.16	1.14	0.03	0.062	1283	3.14	198.36	118.24
DSIN	DMU17	7.52	1.21	0.84	0.97	0.03	0.039	1222	1.8	174.39	94.68
ROZD	DMU18	8.84	1.01	0.95	0.28	0.07	0.064	131	1.46	26.37	286.26
AMIN	DMU19	5.73	0.97	1.45	0.74	0.04	0.159	696	4.15	163.71	230.03
DZAH	DMU20	5.4	0.95	2.83	1.2	0.07	0.044	2699	2.35	44.51	129.27
ABDI	DMU21	10.22	0.6	4.81	0.59	0.03	0.0201	404	2.21	181.41	83.42
ALBZ	DMU22	6.9	1	1.93	1.41	0.03	0.117	418	1.49	228.42	104.07
DSOB	DMU23	6.75	1.06	1.57	1.46	0.03	0.118	655	2.65	221.73	104.58

In order to identify the worst solution for the first six minimization objective functions and the next four maximization objective functions, the process is directed by Table 11 (balance table and fuzzy membership functions for objective functions). In particular, the best x_i_ solution for each objective function should be used to evaluate other objective functions, as presented in Table 11. The worst solution is considered Z_NIS_ for the minimization objectives_,_ which is the highest value of each function. For the maximization objectives, the minimum value of each Index is considered the worst solution of Z_NIS_.

**Table 11:** Balance table and fuzzy membership functions for objective functions.

$X_{i}^{*}$	Goal Direction	$Z_{1}$	$Z_{2}$	$Z_{3}$	$Z_{4}$	$Z_{5}$	$Z_{6}$	$Z_{7}$	$Z_{8}$	$Z_{9}$	$Z_{10}$
$X_{1}^{*}$	Min $Z_{1}$	5.4	0.95	2.83	1.2	0.07	0.044	2699	2.35	44.51	129.27
$X_{2}^{*}$	Min $Z_{2}$	13.38	0.49	3.87	0.7	0.03	0.0997	213	2.06	183.48	133.22
$X_{3}^{*}$	Min $Z_{3}$	7.93	7.07	0.1	1.27	0.03	0.0716	1553	1.85	187.62	54.67
$X_{4}^{*}$	Min $Z_{4}$	11.58	0.59	2.85	0.01	0.02	0.0152	799	0.69	110.28	30.16
$X_{5}^{*}$	Min $Z_{5}$	11.58	0.59	2.85	0.01	0.02	0.0152	799	0.69	110.28	30.16
$X_{6}^{*}$	Min $Z_{6}$	11.58	0.59	2.85	0.01	0.02	0.0152	799	0.69	110.28	30.16
$X_{7}^{*}$	Max $Z_{7}$	7.43	1.18	1.22	1.03	0.02	0.097	3344	1.93	157.67	59.33
$X_{8}^{*}$	Max $Z_{8}$	6.81	0.73	2.28	6.24	0.21	0.106	227	5.74	147.27	438.33
$X_{9}^{*}$	Max $Z_{9}$	8.96	0.97	1.36	1.48	0.03	0.109	121	2.64	228.88	369.42
$X_{10}^{*}$	Max Z_10_	6.81	0.73	2.28	6.24	0.21	0.106	227	5.74	147.27	438.33
$Z_{i}^{*}$(NIS)		13.38	7.07	3.87	6.24	0.21	0.109	121	0.69	44.51	30.16

In general, we can use both deviations (u) and (v) for each case to plan the target. In all situations, we first identify the undesirable deviation of the goal in the target and then try to minimize the same. According to the multi-objective problem where the first six objective functions are minimization and the next four objective functions are maximization, weights have been considered. MCDM, AHP, and TOPSIS methods can also be used to determine the weight of goals and the level of priority. Therefore, determining the weights according to the type of objective function is directly related to achieving the desired results. Information regarding financial indicators are presented in Table 12.

**Table 12:** Information regarding financial indicators.

	Indicator	$\tilde{b}$	u	v	K
I1	Price to earnings ratio per share (P/E)	Uncertainty	1	0	39.41586
I2	Quick ratio	Uncertainty	1	0	8.422589
I3	Debt-to-equity ratio	Uncertainty	1	0	13.33742
I4	Beta index (β) according to the desired industry’s efficiency	Uncertainty	1	0	8.025453
I5	Sigma (∆) index	Uncertainty	1	0	0.267955
I6	Stock price forecast error (RMSE)	Uncertainty	1	0	0.45938
O1	Earnings Per Share (EPS)	Uncertainty	0	1	6929.326
O2	One year return	Uncertainty	0.55	0.45	12.25
O3	Liquidity ratio	Uncertainty	0	1	846.256
O4	Earnings per share growth rate	Uncertainty	0	1	817.1175

In the next step, we define the membership function for each objective function based on the range of changes derived from the balance table for each function’s value.


$$\begin{array}{c} \mu {(b}_{1})=\left\{ \begin{array}{c} \;\;\;1\;\;\;\;\;\;\;\;\;\;\;\;\;\;\;\;\;\;\;\;\;\;\;\;\;\;\;\;\;\;\;\;\;b_{1}\leq 5.4\;\;\;\\ \frac{13.38-b_{1}\;}{7.98}\;\;\;\;\;\;\;\;\;\;5.4\;<\;b_{1}<13.38\\ \;\;\;0\;\;\;\;\;\;\;\;\;\;\;\;\;\;\;\;\;\;\;\;\;\;\;\;\;\;\;\;\;\;b_{1}\geq 13.38\;\; \end{array}\right. \end{array}$$ (33)

$$\begin{array}{c} \mu {(b}_{2})=\left\{ \begin{array}{c} \;\;\;1\;\;\;\;\;\;\;\;\;\;\;\;\;\;\;\;\;\;\;\;\;\;\;\;\;\;\;\;\;\;\;b_{2}\leq 0.49\\ \frac{7.07-b_{2}\;}{6.58}\;\;\;\;\;\;\;\;\;\;\;0.49\;<\;b_{2}<7.07\\ \;\;\;0\;\;\;\;\;\;\;\;\;\;\;\;\;\;\;\;\;\;\;\;\;\;\;\;\;\;\;\;\;\;\;\;b_{2}\geq 7.07\;\; \end{array}\right. \end{array}$$ (34)

$$\begin{array}{c} \mu {(b}_{3})=\left\{ \begin{array}{c} \;\;\;1\;\;\;\;\;\;\;\;\;\;\;\;\;\;\;\;\;\;\;\;\;\;\;\;\;\;\;\;\;\;b_{3}\leq 0.1\\ \frac{3.87-b_{3}\;}{3.77}\;\;\;\;\;\;\;\;\;\;0.1\;<\;b_{3}<3.87\\ \;\;\;0\;\;\;\;\;\;\;\;\;\;\;\;\;\;\;\;\;\;\;\;\;\;\;\;\;\;\;\;\;b_{3}\geq 3.87\;\; \end{array}\right. \end{array}$$ (35)

$$\begin{array}{c} \mu {(b}_{4})=\left\{ \begin{array}{c} \;\;\;1\;\;\;\;\;\;\;\;\;\;\;\;\;\;\;\;\;\;\;\;\;\;\;\;\;\;b_{4}\leq 0.01\;\;\;\;\;\;\;\;\\ \frac{6.24-b_{4}\;}{6.23}\;\;\;\;\;\;\;\;\;\;0.01\;<\;b_{4}<6.24\\ \;\;\;0\;\;\;\;\;\;\;\;\;\;\;\;\;\;\;\;\;\;\;\;\;\;\;\;\;\;\;\;\;\;b_{4}\geq 6.24\;\; \end{array}\right. \end{array}$$ (36)

$$\begin{array}{c} \mu {(b}_{5})=\left\{ \begin{array}{c} \;\;\;1\;\;\;\;\;\;\;\;\;\;\;\;\;\;\;\;\;\;\;\;\;\;\;\;\;\;\;\;\;\;\;b_{5}\leq 0.02\\ \frac{0.21-b_{5}\;}{0.19}\;\;\;\;\;\;\;\;\;\;0.02\;<\;b_{5}<0.21\\ \;\;\;0\;\;\;\;\;\;\;\;\;\;\;\;\;\;\;\;\;\;\;\;\;\;\;\;\;\;\;\;\;\;\;b_{5}\geq 0.21\;\; \end{array}\right. \end{array}$$ (37)

$$\begin{array}{c} \mu {(b}_{6})=\left\{ \begin{array}{c} \;\;\;1\;\;\;\;\;\;\;\;\;\;\;\;\;\;\;\;\;\;\;\;\;\;\;\;b_{6}\leq 0.0152\;\;\;\;\;\;\;\;\;\;\\ \frac{0.109-b_{6}\;}{0.0938}\;\;\;\;\;\;\;\;\;\;0.0152\;<\;b_{6}<0.109\\ \;\;\;0\;\;\;\;\;\;\;\;\;\;\;\;\;\;\;\;\;\;\;\;\;\;\;\;\;\;\;\;\;\;\;\;\;\;\;\;\;b_{6}\geq 0.109\;\; \end{array}\right. \end{array}$$ (38)

$$\begin{array}{c} \mu {(b}_{7})=\left\{ \begin{array}{c} \;\;\;1\;\;\;\;\;\;\;\;\;\;\;\;\;\;\;\;\;\;\;\;\;\;b_{7}\geq 3344\;\;\;\;\;\;\;\\ \frac{b_{7}-121\;}{3223}\;\;\;\;\;\;\;\;\;\;121\;<\;b_{7}<3344\\ \;\;\;0\;\;\;\;\;\;\;\;\;\;\;\;\;\;\;\;\;\;\;\;\;\;b_{7}\leq 121\;\;\;\;\;\;\;\;\;\;\; \end{array}\right. \end{array}$$ (39)

$$\begin{array}{c} \mu {(b}_{8})=\left\{ \begin{array}{c} \;\;\;1\;\;\;\;\;\;\;\;\;\;\;\;\;\;\;\;\;\;\;\;\;\;\;\;\;\;\;\;\;\;\;\;\;b_{8}\geq 5.74\\ \frac{b_{8}-0.69\;}{5.05}\;\;\;\;\;\;\;\;\;\;\;0.69\;<\;b_{8}<5.74\\ \;\;\;0\;\;\;\;\;\;\;\;\;\;\;\;\;\;\;\;\;\;\;\;\;\;\;\;\;\;\;\;\;\;\;\;\;b_{8}\leq 0.69\;\; \end{array}\right. \end{array}$$ (40)

$$\begin{array}{c} \mu {(b}_{9})=\left\{ \begin{array}{c} \;\;\;1\;\;\;\;\;\;\;\;\;\;\;\;\;\;\;\;\;\;\;\;\;\;\;\;\;\;\;\;\;\;\;\;\;\;\;\;\;\;\;b_{9}\geq 228.88\\ \frac{b_{9}-44.51\;}{184.37}\;\;\;\;\;\;\;\;\;\;\;\;\;44.51\;<\;b_{9}<228.88\\ \;\;\;0\;\;\;\;\;\;\;\;\;\;\;\;\;\;\;\;\;\;\;\;\;\;\;\;\;\;\;\;\;\;\;\;\;\;\;\;\;\;\;\;\;\;b_{9}\leq 44.51\;\; \end{array}\right. \end{array}$$ (41)

$$\begin{array}{c} \mu {(b}_{10})=\left\{ \begin{array}{c} \;\;\;1\;\;\;\;\;\;\;\;\;\;\;\;\;\;\;\;\;\;\;\;\;\;\;\;\;\;\;\;\;\;\;\;b_{10}\geq 438.33\;\;\;\;\;\;\;\;\\ \frac{b_{10}-30.16\;}{408.17}\;\;\;\;\;\;\;\;\;\;\;\;\;30.16\;<\;b_{10}<438.33\\ \;\;\;0\;\;\;\;\;\;\;\;\;\;\;\;\;\;\;\;\;\;\;\;\;\;\;\;\;\;\;\;\;\;\;\;\;\;\;\;\;\;\;\;\;b_{10}\leq 30.16\;\;\; \end{array}\right. \end{array}$$ (42)

Following this, we transform the initial multi-objective model into an equivalent single-objective model using an integration function.


Max

$$\begin{array}{c} s.t\;\;\;\;\;\;\;\;\left\{ \begin{array}{c} 0\leq \lambda \leq 1\\ b_{1}+7.98\lambda \;\leq \;\;13.38\\ b_{2}+6.58\lambda \;\leq \;\;7.07 \end{array}\right. \end{array}$$ (43)

$$\begin{array}{c} b_{3}+3.77\lambda \;\leq \;\;3.87 \end{array}$$

$$\begin{array}{c} b_{4}+6.23\lambda \;\leq \;\;6.24 \end{array}$$

$$\begin{array}{c} b_{5}+0.19\lambda \;\leq \;\;0.21 \end{array}$$

$$\begin{array}{c} b_{6}+0.0938\lambda \;\leq \;\;0.109 \end{array}$$

$$\begin{array}{c} b_{7}-3223\;\geq \;121 \end{array}$$

$$\begin{array}{c} b_{8}-5.05\lambda \;\geq \;\;0.69 \end{array}$$

$$\begin{array}{c} b_{9}-184.37\lambda \;\geq \;\;44.51 \end{array}$$

$$\begin{array}{c} b_{10}-408.17\lambda \;\geq \;\;30.16 \end{array}$$

After solving this model in the GAMS software, the optimal values (b) for each objective function are obtained and then introduced into the main model.

Another variation of fuzzy goal programming can be introduced based on the following approach: By finding the optimal and the worst-case scenario for each of the objective functions, we use the variable λ. Since each objective function can have a minimizing or maximizing nature and each has a corresponding financial index, we set the λ value between 0 and 1. This approach assists us in determining the ideal values for each Index.


$$\begin{array}{c} b_{1}=5.4\lambda +\;13.38(1-\lambda ) \end{array}$$ (44)

$$\begin{array}{c} b_{2}=0.49\lambda +\;7.07(1-\lambda ) \end{array}$$

$$\begin{array}{c} b_{3}=0.1\lambda +\;3.87(1-\lambda ) \end{array}$$

$$\begin{array}{c} b_{4}=0.01\lambda \;+\;6.24(1-\lambda ) \end{array}$$

$$\begin{array}{c} b_{5}=0.02\lambda +\;0.21(1-\lambda ) \end{array}$$

$$\begin{array}{c} b_{6}=0.0152\lambda +\;0.109(1-\lambda ) \end{array}$$

$$\begin{array}{c} b_{7}=121(1-\lambda )+\;3344\; \end{array}$$

$$\begin{array}{c} b_{8}=0.69(1-\lambda )+\;5.74\lambda \end{array}$$

$$\begin{array}{c} b_{9}=44.51(1-\lambda )+\;228.88\lambda \end{array}$$

$$\begin{array}{c} b_{10}=30.16(1-\lambda )+\;438.33\lambda \end{array}$$

$$\begin{array}{c} 0\leq \;\leq 1 \end{array}$$

In the relationships presented in Table 13, the ideal values of each objective function have been determined for different values of λ. The final results of the model, solved and coded in GAMS software, are shown in the following tables for various λ values and their corresponding ideals. It’s important to note that using a strict model (λ = 0) will yield the ideal values, while a less stringent approach (λ = 1) will also find ideal values.

**Table 13:** Ideal values of each objective function corresponding to different landa (□) values.

$b_{i}$	λ				
	λ = 0	λ = 0/25	λ = 0/5	λ = 0/75	λ = 1
$b_{I1}$	13.38	11.385	9.39	7.395	5.4
$b_{I2}$	7.07	5.425	3.78	2.135	0.49
$b_{I3}$	3.87	2.9275	1.985	1.0425	0.1
$b_{I4}$	6.24	4.6825	3.125	1.5675	0.01
$b_{I5}$	0.21	0.1625	0.115	0.0675	0.02
$b_{I6}$	0.109	0.08555	0.0621	0.03865	0.0152
$b_{O1}$	121	926.75	1732.5	2538.25	3344
$b_{O2}$	0.69	1.9525	3.215	4.4775	5.74
$b_{O3}$	44.51	90.6025	136.695	182.7875	228.88
$b_{O4}$	30.16	132.2025	234.245	336.2875	438.33

Goal programming is a multicriteria decision-making technique and a subset of multi-objective decision-making. It can be viewed as an extension of linear programming that accommodates multiple objectives and is designed to achieve expected values for these objectives. However, the statement about goal programming being “specifically linear when it has only one objective” is incorrect and should be removed, as this would simply be linear programming. Goal programming is a multicriteria decision-making model in the field of linear algebra. This model encompasses multiple objectives simultaneously and is developed to minimize deviation from the objectives. The core strength of goal programming is that it considers the constraints and goals with decision variables; it also eliminates and lessens weak human reasoning during programming and decision-making. This strength becomes notably apparent when we seek to optimize multiple factors simultaneously.

The GP method is used for the relative fulfillment of goals, meaning the decision-maker cannot fulfill them as they desire. Therefore, in such decisions, the decision-maker tries to obtain a solution closest to the goals in mind.

GP was initially introduced in 1955 in a paper by Charnes, Cooper, and Fergusen as a method of calculating losses. After some time, in 1961, Charnes and Cooper proposed a more precise definition, leading to the emergence of the term “goal programming” for the first time. In the mid-70s, the application of GP in articles increased. It can be said with certainty that GP is still one of the most widely used techniques in multicriteria decision-making in numerous cases.

In this model, i denotes the number of objective functions, a_ij_x_j_ linear function of x, and b_i_ the level of value or goal for the function. n_i_ and p_i_ denote negative and positive deviations from the goal. u_i_ and v_i_ denote the non-negative weight assigned to the diversions, while D represents the maximum diversion that is minimized and is only a part of function Z. K_i_ denotes the normalizing constant for the i-th objective function, and a_ij_ is the index rate of i for asset j, and x_j_ is the selected amount to purchase asset j.

Min Z = D


$$s.t.\left\{ \begin{array}{c} \frac{1}{k_{i}}\;(u_{i}n_{i}+\;v_{i}p_{i})\leq D\;,\;\forall_{i}\\ \sum_{j=1}^{n}a_{ij}X_{j}+\;n_{i}-p_{i}=\;{\tilde{b}}_{i}\;,\;\forall_{i}\;\\ \sum_{j=1}^{n}X_{j}=1\;0\leq \;X_{j}\;\leq 1 \end{array}\right.\;$$ (45)

All $n_{i},p_{i\;}\;variables\;\geq \;0\;,\;\forall_{i}\;$

Min Z = D


$$S.t:\;\left\{ \begin{array}{c} \frac{1}{k_{I1}}\;(u_{I1}n_{I1}+\;v_{I1}p_{I1})\leq D\\ \frac{1}{k_{I2}}\;\left(u_{I2}n_{I2}+\;v_{I2}p_{I2}\right)\leq D\\ \frac{1}{k_{I3}}\;\left(u_{I3}n_{I3}+\;v_{I3}p_{I3}\right)\leq D \end{array}\right.\;$$

$$\frac{1}{k_{I4}}\;\left(u_{I4}n_{I4}+\;v_{I4}p_{I4}\right)\leq D\;$$

$$\frac{1}{k_{I5}}\;\left(u_{I5}n_{I5}+\;v_{I5}p_{I5}\right)\leq D\;$$

$$\;\frac{1}{k_{I6}}\;\left(u_{I6}n_{I6}+\;v_{I6}p_{I6}\right)\leq D\;$$

$$\frac{1}{k_{O1}}\;\left(u_{O1}n_{O1}+\;v_{O1}p_{O1}\right)\leq D\;$$

$$\frac{1}{k_{O2}}\;\left(u_{O2}n_{O2}+\;v_{O2}p_{O2}\right)\leq D\;$$

$$\frac{1}{k_{O3}}\;\left(u_{O3}n_{O3}+\;v_{O3}p_{O3}\right)\leq D\;$$

$$\frac{1}{k_{O4}}\;\left(u_{O4}n_{O4}+\;v_{O4}p_{O4}\right)\leq D\;$$

$$\sum_{j=1}^{n}{I1}_{j}X_{j}+\;n_{I1}-p_{I1}=\;b_{I1}\;$$ (46)

$$\sum_{j=1}^{n}{I2}_{j}X_{j}+\;n_{I2}-p_{I2}=\;b_{I2}\;$$

$$\sum_{j=1}^{n}{I3}_{j}X_{j}+\;n_{I3}-p_{I3}=\;b_{I3}\;$$

$$\sum_{j=1}^{n}{I4}_{j}X_{j}+\;n_{I4}-p_{I4}=\;b_{I4}\;$$

$$\sum_{j=1}^{n}{I5}_{j}X_{j}+\;n_{I5}-p_{I5}=\;b_{I5}\;$$

$$\sum_{j=1}^{n}{I6}_{j}X_{j}+\;n_{I6}-p_{I6}=\;b_{I6}\;$$

$$\sum_{j=1}^{n}{O1}_{j}X_{j}+\;n_{O1}-p_{O1}=\;b_{O1}\;$$

$$\sum_{j=1}^{n}{O2}_{j}X_{j}+\;n_{O2}-p_{O2}=\;b_{O2}\;$$

$$\sum_{j=1}^{n}{O3}_{j}X_{j}+\;n_{O3}-p_{O3}=\;b_{O3}\;$$

$$\sum_{j=1}^{n}{O4}_{j}X_{j}+\;n_{O4}-p_{O4}=\;b_{O4}\;$$

$$\;\sum_{j=1}^{n}X_{j}=1\;0\leq \;X_{j}\;\leq 1$$

$$All\;n_{i},p_{i\;}\;variables\;\geq \;0\;,\;\forall_{i}\;$$

### 4.6. Analyzing the results of stock portfolio optimization using the fuzzy goal programming method

After coding and inputting of the model and data into the GAMS software, the model was executed, and the results are displayed in the tables below. The quantity of each stock selected for purchase was determined to meet the desired indicators. By varying the λ values and specifying the goal for each indicator, the outcomes are detailed separately in the Tables 14–18.

**Table 14:** The quantity of selected shares with λ = 0.

The quantity of selected shares X_j_	
X_7_	0.528
X_15_	0.472

**Table 15:** The quantity of selected shares with λ = 0.25.

The quantity of selected shares X_j_	
X_7_	0.516
X_15_	0.484

**Table 16:** The quantity of selected shares with λ = 0.5.

The quantity of selected shares X_j_	
X_2_	0.117
X_7_	0.438
X_15_	0.414
X_18_	0.001
X_20_	0.030

**Table 17:** The quantity of selected shares with λ = 0.75.

The quantity of selected shares X_j_	
X_2_	0.020
X_6_	0.090
X_7_	0.234
X_15_	0.656

**Table 18:** The quantity of selected shares with λ = 1.

The quantity of selected shares X_j_	
X_15_	1

By changing Landa between zero and one, different values are determined for the goal of each Index, and by solving the model with each of the values in the software, the selected value of each share is determined to achieve the desired goals. According to the outputs of the third stage of modeling, if we want to take the easy model and obtain the requirements of the objective functions more optimally, the values obtained from λ equal to one we select consider stocks to buy; otherwise, if we want to take the strict model, the values obtained from λ equal to zero we select consider stocks to buy, and if we want to have a middle state between the two mentioned cases we will use λ values between zero and one to buy selected stocks. Therefore, according to the mentioned cases, the more selected items of each share in terms of λ are equal to one and tend in this direction, the more the requirements of the objective functions will be fulfilled.

## 5. Conclusions and suggestions

### 5.1. Conclusion

In this research, a three-stage model was developed to optimize stock portfolios. Portfolio selection and management are among the most critical decisions in the financial field. This process is highly sensitive to uncontrollable variables, making it crucial for investors who must allocate their budgets across financial assets within their portfolios. Financial analysts face several major challenges, including identifying factors that influence investor decisions, quantifying these factors, and understanding how they impact portfolio selection.

To address these challenges, this research proposes a three-stage model. In the first stage, efficient stocks were identified using robust ratio data envelopment analysis (RR-DEA) applied to pharmaceutical industry stocks from the Tehran Stock Exchange (TSE). This analysis accounted for uncertainty in financial indicators and eliminated inefficient stocks. In the second stage, the selected stocks underwent further analysis using the ANFIS algorithm to predict their closing prices for a given period (T). The predictions’ accuracy was evaluated using Root Mean Square Error (RMSE). Finally, in the third stage, the optimization process was carried out by considering the ideal of each Index in a fuzzy manner. The model was run with different ideal values, and the results were presented separately to cater to the decision-maker’s preferences. The selected data in this issue is considered historically, and in the second part, the prediction is made through these data. On the other hand, by changing the 0 ≤ x_j_ ≤ 1 constraint in the FGP section, it is possible to determine the maximum percentage of each share that can be purchased. For example, 0 ≤ x_j_ ≤ 0.25 and 0 ≤ x_j_ ≤ 0.5 and again model and solve the problem. To consider factors such as market conditions, systematic and nonsystematic risks were considered as inputs in the modeling. This comprehensive approach aimed to optimize the stock portfolio by balancing multiple objectives and addressing the inherent uncertainties in financial decision-making. In the end, the main contributions of this study can be summarized as follows:

- The paper introduces a novel RR-DEA method under data uncertainty.
- An ANFIS algorithm is proposed to predict each price per share.
- Fuzzy goal programming method with different goals for each indicator.

### 5.2. Suggestions for future studies

We used the proposed RR-DEA methods as a filter to select efficient stocks in conditions of uncertainty to select effective stocks according to the considered indicators. ANFIS algorithm was used to predict stock prices. Also, the FGP method was used in the conditions of uncertainty in the goal of each index to optimize the stock portfolio, which can be used separately from each of the methods proposed in this article in accordance with the upcoming problem in other researches. We suggest that future studies use other data on the stocks of different industries in other countries. Further, in the first stage, uncertainty can be considered in some ratios and stocks before solving the model. The input parameters of the model can be increased to predict the prices and interest rates in the second stage of modeling and prediction using the enhanced neuro-fuzzy algorithm. In this algorithm, we employed the genfis3 function for prediction in MATLAB. However, future studies can utilize other functions for the same purpose. In the third stage, the model can be introduced as robust modeling by considering uncertainty in data. Methods like Jiménez’s fuzzy optimization can also be employed to manage the uncertainty in the goals of each Index. In the fuzzy goal programming section, the researchers could explore the potential integration of other MCDM techniques, such as the Analytic Hierarchy Process (AHP) or the Technique for Order of Preference by Similarity to Ideal Solution (TOPSIS), to determine the goal weights and priority levels.

## Supporting information

S1 Data Information on the existing stocks of the pharmaceutical industry (March 2013-March 2014) from Tehran Stock Exchange. (DOCX)

S2 Data Data regarding closing prices from 02/03/2013 to 04/02/2018 from the Tehran Stock Exchange website as a time series for all the selected stocks. (XLSX).
