# Supplementary material for: Novel methods for selecting stock portfolio in conditions of uncertainty and forecasting with RR-DEA, ANFIS, FGP: A case study of Tehran stock exchange
Source: PLoS One. 2025 Jul 15;20(7):e0321370. doi: 10.1371/journal.pone.0321370 (PMC12262894; doi:10.1371/journal.pone.0321370)
Supplement: S1 Data — (DOCX) [file pone.0321370.s001.docx]

**Information on the existing stocks of the pharmaceutical industry (March 2013-March 2014) from Tehran Stock Exchange**

| **Symbol** | **DMU** | **I(1)** | **I(2)** | **I(3)** | **I(4)** | **I(5)** | **O(1)** | **O(2)** | **O(3)** | **O(4)** |
| --- | --- | --- | --- | --- | --- | --- | --- | --- | --- | --- |
| **PDRO** | DMU01 | 7.43 | 1.18 | 1.22 | 1.03 | 0.02 | 3344 | 1.93 | 157.67 | 59.33 |
| **DLGM** | DMU02 | 13.38 | 0.49 | 3.87 | 0.7 | 0.03 | 213 | 2.06 | 183.48 | 133.33 |
| **THSH** | DMU03 | 11.58 | 0.59 | 2.85 | 0.01 | 0.02 | 799 | 0.69 | 110.28 | 30.16 |
| **DDPK** | DMU04 | 7.7 | 0.86 | 2.27 | 0.54 | 0.05 | 693 | 2.73 | 122.76 | 56.85 |
| **TMVD** | DMU05 | 6.58 | 1.16 | 1 | 0.64 | 0.02 | 2965 | 1.04 | 166.99 | 10.66 |
| **DAML** | DMU06 | 8.7 | 0.87 | 3.91 | 0.57 | 0.03 | 1386 | 1.98 | 156.08 | 2.74 |
| **DFRB** | DMU07 | 7.76 | 1.07 | 1.84 | 1.4 | 0.03 | 1277 | 2.04 | 164.07 | 31.17 |
| **DKSR** | DMU08 | 8.96 | 0.97 | 1.36 | 1.48 | 0.03 | 121 | 2.64 | 228.88 | 369.42 |
| **DARO** | DMU09 | 7.93 | 7.07 | 0.1 | 1.27 | 0.03 | 1553 | 1.85 | 187.63 | 54.67 |
| **DABO** | DMU10 | 9.03 | 0.86 | 3.44 | 0.71 | 0.03 | 1357 | 2.3 | 143.68 | 93.15 |
| **DRZK** | DMU11 | 7.91 | 0.96 | 1.72 | 0.68 | 0.03 | 1493 | 2.88 | 167.43 | 96.65 |
| **DOSE** | DMU12 | 18.43 | 1.06 | 1.23 | 1.56 | 0.04 | 997 | 1.92 | 169.7 | 67 |
| **PKSH** | DMU13 | 6.41 | 0.9 | 5.95 | 1.67 | 0.03 | 528 | 0.73 | 227.86 | 53.22 |
| **IRDR** | DMU14 | 7.47 | 0.72 | 3 | 1.09 | 0.03 | 306 | 1.59 | 187.99 | 230.39 |
| **DALZ** | DMU15 | 7.46 | 1.28 | 1.21 | 1.49 | 0.03 | 956 | 2.49 | 205.22 | 111.3 |
| **DSBH** | DMU16 | 8.39 | 1.35 | 0.86 | 1.6 | 0.04 | 2340 | 2.91 | 155.82 | 95.56 |
| **DPAK** | DMU17 | 6.82 | 0.79 | 4.43 | 1.3 | 0.05 | 666 | 2.52 | 177.08 | 119.82 |
| **DJBR** | DMU18 | 6.94 | 1.21 | 0.94 | 0.94 | 0.03 | 659 | 3.14 | 219.36 | 122.76 |
| **KIMI** | DMU19 | 6.81 | 0.73 | 2.28 | 6.24 | 0.21 | 227 | 5.74 | 147.27 | 438.33 |
| **EXIR** | DMU20 | 8.2 | 0.82 | 5.16 | 1.14 | 0.03 | 1283 | 3.14 | 198.36 | 118.24 |
| **DSIN** | DMU21 | 7.52 | 1.21 | 0.84 | 0.97 | 0.03 | 1222 | 1.8 | 174.39 | 94.68 |
| **ROZD** | DMU22 | 8.84 | 1.01 | 0.95 | 0.28 | 0.07 | 131 | 1.46 | 26.37 | 286.26 |
| **AMIN** | DMU23 | 5.73 | 0.97 | 1.45 | 0.74 | 0.04 | 696 | 4.15 | 163.71 | 230.03 |
| **DZAH** | DMU24 | 5.4 | 0.95 | 2.83 | 1.2 | 0.07 | 2699 | 2.35 | 44.51 | 129.27 |
| **ABDI** | DMU25 | 10.22 | 0.6 | 4.81 | 0.59 | 0.03 | 404 | 2.21 | 181.41 | 83.42 |
| **ALBZ** | DMU26 | 6.9 | 1 | 1.93 | 1.41 | 0.03 | 418 | 1.49 | 228.42 | 104.07 |
| **DSOB** | DMU27 | 6.75 | 1.06 | 1.57 | 1.46 | 0.03 | 655 | 2.65 | 221.73 | 104.58 |

| **Input Variables** | **Symbol** | **Output Variables** | **Symbol** |
| --- | --- | --- | --- |
| The price-to-earnings (P/E) ratio per share  Quick ratio  Debt-to-equity ratio  The Beta (β) Index is based on the industry's returns.  The sigma index | I(1)  I(2)  I(3)  I(4)  I(5) | Earnings per share (EPS)  One-year returns  Liquidity ratio  Earnings per share (EPS) growth rate | O(1)  O(2)  O(3)  O(4) |
